# Human HDL subclasses modulate energy metabolism in skeletal muscle cells

**DOI:** 10.1016/j.jlr.2023.100481

**Published:** 2023-11-24

**Authors:** Jenny Lund, Emilia Lähteenmäki, Tiia Eklund, Hege G. Bakke, G. Hege Thoresen, Eija Pirinen, Matti Jauhiainen, Arild C. Rustan, Maarit Lehti

**Affiliations:** 1 Section for Pharmacology and Pharmaceutical Biosciences, Department of Pharmacy, University of Oslo, Oslo, Norway; 2 Faculty of Sport and Health Sciences, University of Jyväskylä, Jyväskylä, Finland; 3 Department of Biological and Environmental Science, University of Jyväskylä, Jyväskylä, Finland; 4 Department of Pharmacology, Institute of Clinical Medicine, University of Oslo, Oslo, Norway; 5 Research Program for Clinical and Molecular Metabolism, Faculty of Medicine, University of Helsinki, Helsinki, Finland; 6 Research Unit for Biomedicine and Internal Medicine, Faculty of Medicine, University of Oulu, Oulu, Finland; 7 Medical Research Center Oulu, Oulu University Hospital and University of Oulu, Oulu, Finland; 8 Biocenter Oulu, University of Oulu, Oulu, Finland; 9 Department of Public Health and Welfare, Minerva Foundation Institute for Medical Research and Finnish Institute for Health and Welfare, Helsinki, Finland

**Keywords:** cellular respiration, substrate oxidation, glycolysis, oxidative phosphorylation, skeletal muscle myotubes, HDL subclasses, fatty acid/transport, glucose, lipoproteins/metabolism, mitochondria, lipoproteins/receptors

## Abstract

In addition to its antiatherogenic role, HDL reportedly modulates energy metabolism at the whole-body level. HDL functionality is associated with its structure and composition, and functional activities can differ between HDL subclasses. Therefore, we studied if HDL_2_ and HDL_3_, the two major HDL subclasses, are able to modulate energy metabolism of skeletal muscle cells. Differentiated mouse and primary human skeletal muscle myotubes were used to investigate the influences of human HDL_2_ and HDL_3_ on glucose and fatty uptake and oxidation. HDL-induced changes in lipid distribution and mRNA expression of genes related to energy substrate metabolism, mitochondrial function, and HDL receptors were studied with human myotubes. Additionally, we examined the effects of apoA-I and discoidal, reconstituted HDL particles on substrate metabolism. In mouse myotubes, HDL subclasses strongly enhanced glycolysis upon high and low glucose concentrations. HDL_3_ caused a minor increase in ATP-linked respiration upon glucose conditioning but HDL_2_ improved complex I–mediated mitochondrial respiration upon fatty acid treatment. In human myotubes, glucose metabolism was attenuated but fatty acid uptake and oxidation were markedly increased by both HDL subclasses, which also increased mRNA expression of genes related to fatty acid metabolism and HDL receptors. Finally, both HDL subclasses induced incorporation of oleic acid into different lipid classes. These results, demonstrating that HDL subclasses enhance fatty acid oxidation in human myotubes but improve anaerobic metabolism in mouse myotubes, support the role of HDL as a circulating modulator of energy metabolism. Exact mechanisms and components of HDL causing the change, require further investigation.

## Graphical abstract

**Figure:**
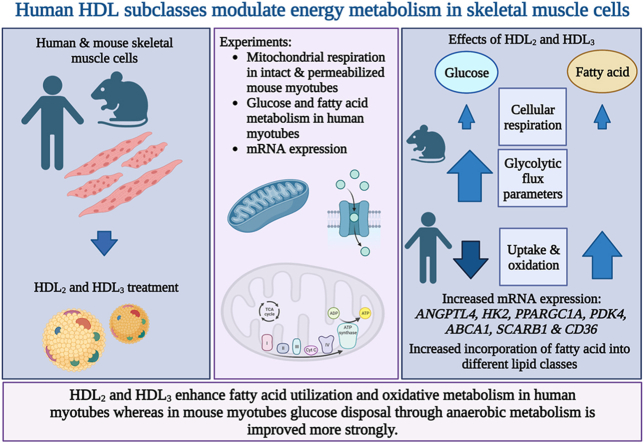

HDL is known as the only circulating antiatherogenic lipoprotein class that transports excess cholesterol from peripheral tissues to the liver in a process called reverse cholesterol transport, which is currently considered its major atheroprotective function (1). In addition, other cardio-metabolically beneficial functions of HDL have been identified such as antioxidative, anti-inflammatory, and vasodilatory activity as well as the regulation of insulin secretion and insulin sensitivity (2, 3, 4). However, HDL particles have a highly heterogenous structure and biological activities that cannot be determined by only measuring plasma HDL cholesterol concentration (5). This has led to the suggestion that HDL quality and functional capacity, rather than its concentration, have a more important role in the health beneficial functions of HDL, such as protection from the development of atherosclerosis (5).

HDL functions are associated with particle structure and composition, which are shown as differences in biological activities between two major HDL subclasses, HDL_2_ and HDL_3_ (5, 6). Smaller and denser HDL_3_ particles are more involved in cholesterol efflux process, whereas larger HDL_2_ particles participate more in antithrombotic activities (5). Moreover, diseases and lifestyle factors can modulate the distribution of certain HDL subclasses and their functional capacity (7, 8). Obesity is known to shift HDL particle size distribution toward smaller sizes, resulting in enrichment of the smaller HDL_3_ particles, which is associated with decreased apoA-I abundance in HDL particles and an increased amount of TAGs in HDL via cholesteryl ester transfer protein function (8). This alters the fluidity of HDL particles leading to dysregulation of reverse cholesterol transport, decrease in the activity of antioxidative enzymes, and changes in the function of lipid transfer proteins involved in HDL metabolism (4). In turn, endurance training that develops aerobic capacity and improves cardiometabolic health is known to increase circulating HDL-cholesterol levels and shifting HDL subpopulation toward bigger HDL_2_ particle sizes (9, 10, 11). Based on these observations, HDL subclasses may have different functions, but it is unclear how these are expressed in cellular energy metabolism and mitochondrial functioning.

It has been shown that HDL can modulate whole-body energy metabolism. The infusion of reconstituted HDL in subjects with T2D resulted in increased glucose disposal and inhibition of fasting-induced lipolysis and fatty acid oxidation (FAO) (12, 13). Our previous study using mouse models with either increased HDL level (human apoA-I transgenic [apoA-I tg]) or reduced HDL level (apoA-I deficient [apoA-I KO]) shows that higher circulating HDL level is associated with lower fat mass in body composition and higher control of energy homeostasis turning out as lower fasting glucose and higher glucose tolerance (14). In skeletal muscle, HDL is shown to target several processes of energy metabolism. Glucose uptake and fatty acid (palmitate) oxidation were increased by HDL in primary human myotubes from subjects with T2D (12). Our previous study showed that oxidative metabolism and glycolysis were enhanced by HDL in mouse skeletal muscle cells, suggesting that the effect of HDL was mediated via modulation of mitochondrial function in skeletal muscle (14). However, as HDL particles are very heterogenous and form distinct subclasses with differences in their protein, lipid and cargo content, the different effects, and regulation of cellular energy metabolism and mitochondrial function are currently not known.

To better understand the interaction and differences of HDL subclasses in muscle energy metabolism, we studied the acute effects of human HDL subclasses, HDL_2_ and HDL_3_, on energy metabolism in skeletal muscle cells. Our aim was to evaluate the response of glucose and fatty acid metabolism to treatment with isolated human HDL subclasses. Based on our previous findings with mouse skeletal muscle cells showing increased cellular respiration and glycolysis by HDL (14), we wanted first to investigate this using C2C12 mouse myotubes with similar physiology and genetic background, and then further evaluate with primary human myotubes. We further aimed to examine the human HDL-induced modifications in human myotubes by studying changes in mRNA expression levels of selected genes known to be important in glucose and fatty acid metabolism and for mitochondrial function. Finally, we also investigated the effects of lipid-free apoA-I and discoidal, reconstituted HDL (d-rHDLs) on energy substrate metabolism.

## Materials and methods

### Preparation of lipoproteins and d-rHDL particles

#### Lipoproteins

The two major HDL subclasses, HDL_2_ and HDL_3_, were isolated from the pooled plasma of normolipidemic subjects (plasma obtained from Finnish Red Cross with anticoagulant acid-citrate-dextrose solution; client number 6079; permission number 37/2015) by sequential ultracentrifugation as described by Havel *et al.* (1955) (15). After the isolation of particles containing apoB-100, HDL subclasses including HDL_2_ (1.063 < d < 1.125 g/ml), and HDL_3_ (1.125 < d < 1.210 g/ml) were isolated using a Beckman ultracentrifuge (Ti 50.4 rotor and Ti 50.2 rotor, 259,000 *g* (49,000 rpm) or 218,000 *g* (49,000 rpm) at 10°C). The isolated HDL fractions were immediately dialyzed against PBS (without any cryoprotective agent or EDTA added) prior to the planned experiments. Isolated HDL particles were analyzed for lipids as follows: phospholipids (PL) were analyzed using the PLs B-kit (Wako Chemicals, VA, USA) or Pureauto S PL-kit (Daiichi Pure Chemicals, Tokyo, Japan), triacylglycerols (TAGs) using the triglycerides GPO-PAP kit (Roche Diagnostics, Basel, Switzerland), free cholesterol using the Wako Free Cholesterol C-kit (Wako Chemicals), and total cholesterol using the Cholesterol CHOD-PAP-kit (Roche Diagnostics). Esterified cholesterol was calculated as total cholesterol minus free cholesterol. The amount of protein was determined by the method of Lowry *et al.* using BSA as a standard (16). The means represents mean values calculated from at least 15 different normolipidemic plasma preparations obtained from Finnish Red Cross laboratory. The analyzed total protein and lipid composition of the isolated HDL_2_ particles (as mass%) were as follows: total protein 40%, PLs 28%, cholesteryl esters 25%, free cholesterol 4%, and TAG 3%. Corresponding protein/lipid composition for HDL_3_: total protein 55%, PL 25%, cholesteryl ester 14%, free cholesterol 3%, and TAG 3%. LDL was isolated from the normolipidemic plasma also by using sequential ultracentrifugation (15) with a density cut d = 1.019–1.050 g/ml to avoid contamination of IDL and HDL class particles. For the experiments, the concentration of HDL subclasses and LDL was defined by total protein amount (μg/ml).

#### d-rHDL particles

We also used d-rHDLs, that is, apoA-I-PL-cholesterol d-rHDL particles (apoA-I-PL-C). ApoA-I-PL-C d-rHDLs were prepared with a slight modification of the cholate dialysis method, as described by Jauhiainen and Dolphin (17). The two prepared d-rHDLs consisted of complexes of apoA-I:egg-yolk phosphatidylcholine:cholesterol in the molar ratios of 1:25:5 and 1:250:12.5. In brief, phosphatidylcholine, cholesterol, and apoA-I were incubated in the presence of sodium cholate (55 mmol/l final concentration), 4 ml of 10 mmol/l Tris-HCl (pH 7.5) buffer in a shaking Buchler incubator at 25°C (Haake Buchler Instruments, Saddle Brook, NJ, US) for 20 min. This mixture was then extensively dialyzed against 10 mmol/l Tris-HCl buffer (pH 7.5) for 2–3 days at 4°C to remove inhibitory sodium cholate. ApoA-I-phosphatidylcholine-cholesterol particles were stored at 4°C. We also tested apoA-I alone and lipid-free apoA-I was purified as reported (18).

### Experiments with mouse skeletal muscle cells

#### Cell culturing

The mouse skeletal muscle myoblast C2C12 cell line (ATCC® CRL-1772™, Virginia, US) was used for the cellular respiration measurements. For measurements with the Oxygraph-2k (Oroboros Instruments GmbH, Innsbruck, Austria), myoblasts were seeded in 6-well plates (150,000 cells/well) and cultured in DMEM with high glucose (25 mmol/l) (Lonza, Walkersville, US) supplemented with 10% FBS (Thermo Fisher Scientific), 100 μg/ml penicillin-streptomycin (Thermo Fisher Scientific), and 2 mmol/l L-glutamine (Lonza). When confluency was 80%–95%, medium was replaced with DMEM (25 mmol/l glucose) supplemented with 2% horse serum (Thermo Fisher Scientific), 100 μg/ml penicillin-streptomycin and 2 mmol/l L-glutamine to initiate differentiation into myotubes. The cells were differentiated for 5–6 days and during that period the medium was changed every second day. For measurements with Seahorse XFe96 Analyzer (Agilent, Santa Clara, CA, US), myoblasts were seeded in Seahorse XF96 Cell Culture Microplates (3,500 cells/well) and the differentiation was initiated when confluency reached 70%–80%. The same media and supplements were used as described above. The cells were differentiated for 5–8 days and during that period the medium was changed every third day. Cells were kept in a humidified CO_2_ atmosphere at 37°C throughout the culturing and differentiation and were used until passage number 10.

#### Measurement of cellular respiration and glycolytic flux parameters in intact mouse myotubes

For assessing cellular respiration and glycolytic flux parameters in intact mouse myotubes, oxygen consumption rate (OCR) and extracellular acidification rate (ECAR) were measured with Seahorse XFe96 Analyzer (Agilent). In high glucose conditioning, myotubes were incubated for 3 h (37°C, 5% CO_2_) in DMEM with high glucose (25 mmol/l) supplemented with 2 mmol/l Glutamax™ (Gibco, Paisley, UK) and treated with 20, 50, 100, or 200 μg/ml HDL_2_ or HDL_3_ (as total HDL protein). Controls were medium without lipoproteins and 100 μg/ml LDL (as total LDL protein). After 3 h, the medium was changed to Seahorse XF DMEM (Agilent) supplemented with the following Seahorse XF (Agilent) reagents: 2 mmol/l L-glutamine, 1 mmol/l pyruvate, and 25 mmol/l glucose. Myotubes were incubated 1 h at 37°C, 0.04% CO_2_ with the same HDL treatments and controls as described above.

For fatty acid conditioning, myotubes were incubated for 3 h (37°C, 5% CO_2_) in DMEM with low glucose (5.5 mmol/l) supplemented with 2 mmol/l Glutamax™, 100 μmol/l albumin-bound oleic acid (Sigma-Aldrich) and with the same HDL treatments and controls as for glucose measurements. After 3 h, the medium was changed to Seahorse XF DMEM supplemented with 100 μmol/l albumin-bound oleic acid and the following Seahorse XF (Agilent) reagents: 2 mmol/l L-glutamine, 1 mmol/l pyruvate, and 5.5 mmol/l glucose, again with the same HDL treatments and controls as described above. Myotubes were incubated for 1 h at 37°C, 0.04% CO_2_.

OCR and ECAR were measured simultaneously by using a Seahorse XFe96 Analyzer and Wave 2.6.3 program (Agilent). Four substrate and inhibitor injections were performed to determine several respiration and glycolytic states. All substrates and inhibitors were provided by Sigma-Aldrich and prepared as described on the website of Oroboros Instruments. For OCR, basal respiration was measured before any injections. The first injection, oligomycin (1 μmol/l), was added to inhibit ATP synthase and induce proton leak. Secondly, carbonyl cyanide-p-trifluoromethoxyphenylhydrazone (FCCP) (2 μmol/l) was injected to obtain maximal respiration. The third injection was a combination of rotenone (0.5 μmol/l) and antimycin A (0.5 μmol/l) to inhibit electron transport chain function to determine nonmitochondrial respiration and it was subtracted from OCR respiration states. Finally, 2-deoxy-D-glucose (50 mmol/l) was injected to inhibit glycolysis. ATP-linked respiration was determined by subtracting proton leak from basal respiration. Spare capacity, which illustrates the capacity of cells to respond to increased energy demand, was calculated by subtracting basal respiration from maximal respiration.

For ECAR measurements, basal glycolysis was measured before any injections and glycolytic capacity was determined after oligomycin injection. Glycolytic reserve was defined by subtracting basal glycolysis from glycolytic capacity. Nonglycolytic acidification was determined after a 2-deoxy-D-glucose injection and was subtracted from other glycolytic states.

Because albumin-bound oleic acid partly binds to FCCP and prevents its effect, the concentration of FCCP was optimized to 18 μmol/l in fatty acid measurements. Values were normalized to protein amount estimated by using Pierce BCA Protein Assay Kit (Thermo Fisher Scientific).

#### Cellular respiration measurements with permeabilized mouse myotubes

Before actual respirometry measurements, mouse myotubes were incubated for 4 h (37°C, 5% CO_2_) in DMEM containing 100 μg/ml of HDL_2_, HDL_3_ (as total HDL protein), or sterile H_2_O. For measuring glucose oxidative metabolism, DMEM with 25 mmol/l glucose and no L-glutamine was used, whereas for FAO measurements, DMEM without glucose (Thermo Fisher Scientific) supplemented with 0.5 mmol/l L-carnitine (Sigma-Aldrich) was used. After incubation with HDL subclasses, myotubes were harvested in 6 ml mitochondrial respiration medium 05 (19) and transferred to the chambers of a high-resolution Oxygraph-2k respirometer (Oroboros Instruments GmbH). Then, 100 μg/ml HDL_2_, HDL_3_, or sterile H_2_O were added to the suspensions in the chambers. Measurements were performed at 37°C in closed chambers. The data were collected using DatLab software version 7.3.0.3 (Oroboros Instruments, Innsbruck, Austria).

At the beginning of the measurements, the oxygen concentration of myotube suspensions in the chambers was increased up to 400 μmol/l by using pure oxygen (Oy AGA Ab, Espoo, Finland) to prevent oxygen limitation of respiration and oxygen diffusion. Substrate-uncoupler-inhibitor titration protocols (SUITs) were found at Oroboros Ecosystem SUITbrowser (20). All the used substrates and inhibitors were prepared according to the instructions of Oroboros Instruments and were purchased from Sigma-Aldrich except MgCl_2_ from Merck (Darmstadt, Germany).

The measurements of glucose oxidative metabolism without glycolysis in permeabilized myotubes were performed using the SUIT-008 O2 ce-pce D025 protocol (21) to determine four respiration states. After adding the cells to the chambers, permeabilization was achieved by using an optimal concentration of digitonin (14.2 μmol/l). Pyruvate (5 mmol/l) and malate (2 mmol/l) were added, and the nonphosphorylating leak state (LEAK) was measured. Next injection was ADP + Mg^2+^ (5 mmol/l + 3 mmol/l) for measurement of respiration coupled to phosphorylation of ADP to ATP through complex I (OXPHOS I). Cytochrome c (10 μmol/l) was injected to test the integrity of mitochondrial membranes. After additions of glutamate (10 mmol/l) and succinate (10 mmol/l), complex I+II (OXPHOS I+II) activity was measured. Carbonyl cyanide m-chlorophenyl hydrazone (1 μmol/l per titration) was used to obtain maximal electron transfer state (ETS). Finally, rotenone (0.5 μmol/l) and antimycin A (2.5 μmol/l) were added to inhibit respiratory complexes I and III and shut down the electron transport chain function to determine nonmitochondrial respiration, which was subtracted from respiration states.

FAO measurements in permeabilized myotubes (n = 8) were performed using our own protocol as no appropriate SUIT protocol was available. Again, digitonin (14.2 μmol/l) was used to permeabilize the myotubes. Malate (0.1 mmol/l) was injected to ensure tricarboxylic cycle function and LEAK respiration was measured. Separate injections, ADP + Mg^2+^ (5 mmol/l + 3 mmol/l), and cytochrome c (10 μmol/l) were added to confirm the integrity of mitochondrial membranes. After the addition of palmitoylcarnitine (10 μmol/l), which was used as a fatty acid substrate, complex I related FAO (CI + FAO) was measured. Carbonyl cyanide m-chlorophenyl hydrazone (0.5 μmol/l per titration) was added to induce maximal ETS and finally, rotenone (0.5 μmol/l) and antimycin A (2.5 μmol/l) were injected to shut down electron transport chain function.

Cellular respiration results were normalized to citrate synthase activity from 400 μl subsample taken before the measurements and stored at −80°C. Subsamples were let to melt at room temperature and lysed by freezing them twice with liquid nitrogen. After lysis, subsamples were centrifuged 13,870 *g* for 10 min at 4°C to remove debris. The supernatant was collected, mixed, and spun before citrate synthase activity measurement, which was performed with Konelab 20XTi (Thermo Fisher Scienti, Vantaa, Finland) and Citrate Synthase Assay Kit (Sigma-Aldrich).

#### Coupling control efficiencies and flux control ratios

Three coupling-control efficiencies and flux control ratios (FCRs) were calculated from mitochondrial respiration data of intact and permeabilized myotubes (22). Coupling-control efficiencies and FCRs are internal normalizations of mitochondrial respiration. FCRs illustrate cellular respiration states in relation to a reference state, whereas coupling-control efficiencies express changes in respiration normalized to reference state (22). For intact myotubes, R-L control efficiency ((R-L)/R), E-L coupling control ((E-L)/E), and E-R control efficiency ((E-R)/E) and FCRs including L/R coupling-control ratio, L/E coupling-control ratio, and R/E control ratio were calculated by using basal respiration (R), proton leak (L), and maximal respiration (E) values. For permeabilized myotubes, the same equations were used for OXPHOS (P = R), LEAK (L), and ETS (E).

### Experiments with human skeletal muscle cells

#### Ethics statement

The experiments with human muscle biopsies were obtained after informed written consent and were approved by the Regional Committee for Medical and Health Research Ethics South-East, Oslo, Norway (reference number: 2011/2207). The study adhered to the declaration of Helsinki.

#### Cell culturing

The isolation of satellite cells from all muscle biopsies was performed at the same location and by the same trained researchers and has been described previously (23, 24). Multinucleated human myotubes were established by activation and proliferation of satellite cells isolated from *musculus vastus lateralis* from healthy young men. Cells from up to seven donors were used (n = 7). Donor characteristics are presented in Table 1. For proliferation of myoblasts a DMEM-Glutamax™ (5.5 mmol/l glucose; Thermo Fisher Scientific, Waltham, MA, US) medium supplemented with 10% FBS (Thermo Fisher Scientific), 25 mmol/l Hepes (Sigma-Aldrich, St. Louis, MO, US), 25 IU penicillin (Thermo Fisher Scientific), 25 μg/ml streptomycin (Thermo Fisher Scientific), 50 ng/ml gentamicin (Sigma-Aldrich), 1.25 μg/ml amphotericin B (Thermo Fisher Scientific), 10 ng/ml hEGF (Thermo Fisher Scientific), 0.39 μg/ml dexamethasone (Sigma-Aldrich), and 0.05% BSA (Sigma-Aldrich) were used. At approximately 80% confluence the medium was changed to DMEM-Glutamax™ (5.5 mmol/l glucose) supplemented with 2% FBS, 25 IU penicillin, 25 μg/ml streptomycin, 50 ng/ml gentamicin, 1.25 μg/ml amphotericin B, and 25 pmol/l insulin (Novo Nordisk, Bagsvaerd, Denmark) to initiate differentiation into multinucleated myotubes. The cells were allowed to differentiate for 7 days. During the culturing process the muscle cells were incubated in a humidified 5% CO_2_ atmosphere at 37°C, and the medium was changed every 2–3 days. Experiments were performed on cells from passage number 2 to 4.

**Table 1:** Donor characteristics

	Male Donors (n = 7)
Age (years)	27.2 ± 2.4
Body weight (kg)	72.9 ± 4.3
Body mass index (kg/m^2^)	22.3 ± 1.0
fS-glucose (mmol/l)	4.8 ± 0.1
fS-insulin (pmol/l)	32.7 ± 3.1
fS-C-peptide (pmol/l)	288.0 ± 22.1
fS-triacylglycerols (mmol/l)	0.8 ± 0.1
S-cholesterol (mmol/l)	3.8 ± 0.4
S-HDL (mmol/l)	1.4 ± 0.1
S-LDL (mmol/l)	1.9 ± 0.2

#### Glucose and fatty acid substrate oxidation assays

Skeletal muscle cells (7,000 cells/well) were cultured and differentiated in 96-well CellBIND® microplates (Corning, Schiphol-Rijk, the Netherlands) and subjected to energy substrate oxidation assay as previously described (25). To measure glucose oxidation, myotubes were given D-[^14^C(U)]glucose (0.5 μCi/ml, 200 μmol/l; PerkinElmer NEN®, Boston, MA, US) in CO_2_ capturing medium (Dulbecco's PBS with Mg^2+^ and Ca^2+^, supplemented with 10 mmol/l Hepes and 10 μmol/l BSA) with or without lipoprotein (μg/ml) present. To measure FAO, myotubes were given albumin-bound [1–^14^C]oleic acid (0.5 μCi/ml, 100 μmol/l; PerkinElmer NEN®, Boston, MA, US) in a CO_2_-capturing medium (Dulbecco's PBS supplemented with 10 mmol/l Hepes and 1 mmol/l L-carnitine (Sigma-Aldrich)) with or without lipoprotein present. The oleic acid:BSA (fatty acid–free) ratio was 2.5:1 (wt/wt). A 96-well UniFilter®-96 GF/B microplate (PerkinElmer, Shelton, CT, US), activated for capture of CO_2_ by addition of 1 mol/l NaOH, was subsequently mounted on top of the CellBIND® plate and the setup incubated for 4 h at 37°C. ^14^CO_2_ captured in the filter was measured using a 2,450 μβ^2^ scintillation counter (PerkinElmer). After CO_2_ capture, the myotubes were washed twice with PBS and lysed with 0.1 mol/l NaOH before cell-associated radioactivity (CA) was measured by liquid scintillation.

Acid-soluble metabolites (ASMs) in the [1–^14^C]oleic acid medium were measured using a method modified from Skrede *et al.* (26). In brief, 100 μl incubation media were transferred to a Nunc 96-well polystyrene conical bottom microwell plate (Thermo Fisher Scientific), precipitated with 30 μl BSA (6%) and 300 μl ice-cold HClO_4_ (1 mol/l), and centrifuged at 2,100 *g* for 10 min at 4°C. Thereafter, 200 μl of the supernatant was counted by liquid scintillation (Tri-Carb 1900, PerkinElmer). ASMs mainly consist of tricarboxylic acid cycle metabolites and reflect incomplete fatty acid β-oxidation in the mitochondria.

For glucose, the sum of ^14^CO_2_ and remaining CA was taken as a measurement of total cellular uptake of substrate, CO_2_+CA. For oleic acid, the sum of ^14^CO_2_, ASMs, and remaining CA was taken as a measurement of the total cellular uptake of substrate, CO_2_+ASM+CA. Protein levels in the lysate were measured by the Bio-Rad protein assay using a VICTOR™ *X*4 Multilabel Plate Reader (PerkinElmer).

#### Lipid distribution

Cells were cultured in 96-well plates (7,000 cells/well) and were allowed to proliferate for 7 days and differentiate for 6 days. Thereafter, cells were incubated with albumin-bound [1–^14^C]oleic acid (0.5 μCi/ml, 100 μmol/l), with or without HDL_2_ or HDL_3_ (100 μg/ml as total HDL protein) present, for 8 h. After incubation the cells were washed twice with PBS and harvested in 0.1% SDS. Cellular lipids were isolated as previously described. Briefly, lipids were extracted by addition of chloroform:methanol (2:1, v/v) and 0.9% sodium chloride solution (pH 2). The mixture was then separated into two phases and the organic phase was dried under nitrogen and lipids were redissolved in n-hexane. Lipids were separated by TLC, and radioactivity was quantified by liquid scintillation (Packard Tri-Carb 1900 TR, PerkinElmer). The amount of lipids was related to total cell protein concentration determined by the Pierce protein assay using a VICTOR™ *X*4 Multilabel Plate Reader (PerkinElmer).

#### RNA isolation and analysis of gene expression by quantitative real-time PCR

Cells were cultured in 25 cm^2^ NUNC flasks and were allowed to proliferate for 7 days and differentiate for 7 days. Parts of the flasks were treated with HDL_2_ or HDL_3_ (100 μg/ml as total HDL protein) for the last 24 h of the differentiation period, whereas others were treated for only the last 4 h (acute) of the differentiation period. Total RNA was isolated using QIAGEN RNeasyMini Kit (QIAGEN, Venlo, the Netherlands) and reversely transcribed with High-Capacity cDNA Reverse Transcription Kit (Thermo Fisher Scientific) using a PerkinElmer 2720 Thermal Cycler (25°C for 10 min, 37°C for 80 min, and 85°C for 5 min). Primers were designed using Primer Express® (Thermo Fischer Scientific). Quantitative real-time PCR was performed using a StepOnePlus Real-Time PCR system. Target genes were quantified in duplicates carried out in a 25 μl reaction volume. All assays were run for 40 cycles (95°C for 15 s before 60°C for 60 s). Expression levels were normalized to the housekeeping gene ribosomal protein lateral stalk subunit P0 *(RPLP0)*. The housekeeping gene *GAPDH* was also analyzed; there were no differences between normalizing for *RPLP0* or *GAPDH*. Forward and reverse primers were used at a concentration of 30 μmol/l. Primer sequences are presented in the supplementary material (supplemental Table S1).

### Statistics

In mouse myotube experiments, data are presented as means ± SEM relative to basal. The value *n* represents the number of individual experiment wells as cell lineage has been used in these experiments. Statistical comparisons were performed by one-way or two-way ANOVA with Tukey’s post hoc comparison using GraphPad Prism version 9.3.1 for Windows depending on the number of factors. If data were not normally distributed, the Kruskal–Wallis test was applied with Dunn’s multiple comparisons test. A *P*-value ≤ 0.05 was considered statistically significant. All graphs were generated using GraphPad Prism version 9.3.1.

In human myotube experiments, data are presented as means ± SEM in nmol/mg protein or relative to basal. The value *n* represents the number of different donors, each with at least four technical observations. Statistical comparisons on results from human myotubes were performed by two-way ANOVA with Tukey’s post hoc comparison using GraphPad Prism version 9.3.1 for Mac or by linear mixed model analysis (lipid distribution data) using SPSS version 27 (IBM®SPSS® Statistics for Macintosh, Armonk, NY, US). Linear mixed model analysis was used to compare differences between conditions with within-donor variation and simultaneously compare differences between groups with between-donor variation. The linear mixed model analysis includes all observations in the statistical comparison and takes into account that not all observations are independent. A *P*-value ≤ 0.05 was considered statistically significant.

## Results

### Effects of HDL subclasses on energy metabolism in mouse skeletal muscle cells

The acute (4 h) impact of human HDL_2_ and HDL_3_ isolated from normolipidemic donors on glucose and fatty acid metabolism was first examined with mouse myotubes by measuring cellular respiration and glycolytic flux parameters. Concentration-response studies were performed to evaluate the effect of HDL_2_ and HDL_3_ on glucose and fatty acid metabolism. The concentration range from 20 to 200 μg/ml (as total HDL protein) was chosen based on physiological HDL-C and apoA-I levels in human (27).

#### Effects of HDL_2_ and HDL_3_ on glycolytic flux parameters and cellular respiration upon high glucose conditioning in cultured mouse myotubes

**Fig. 1:**
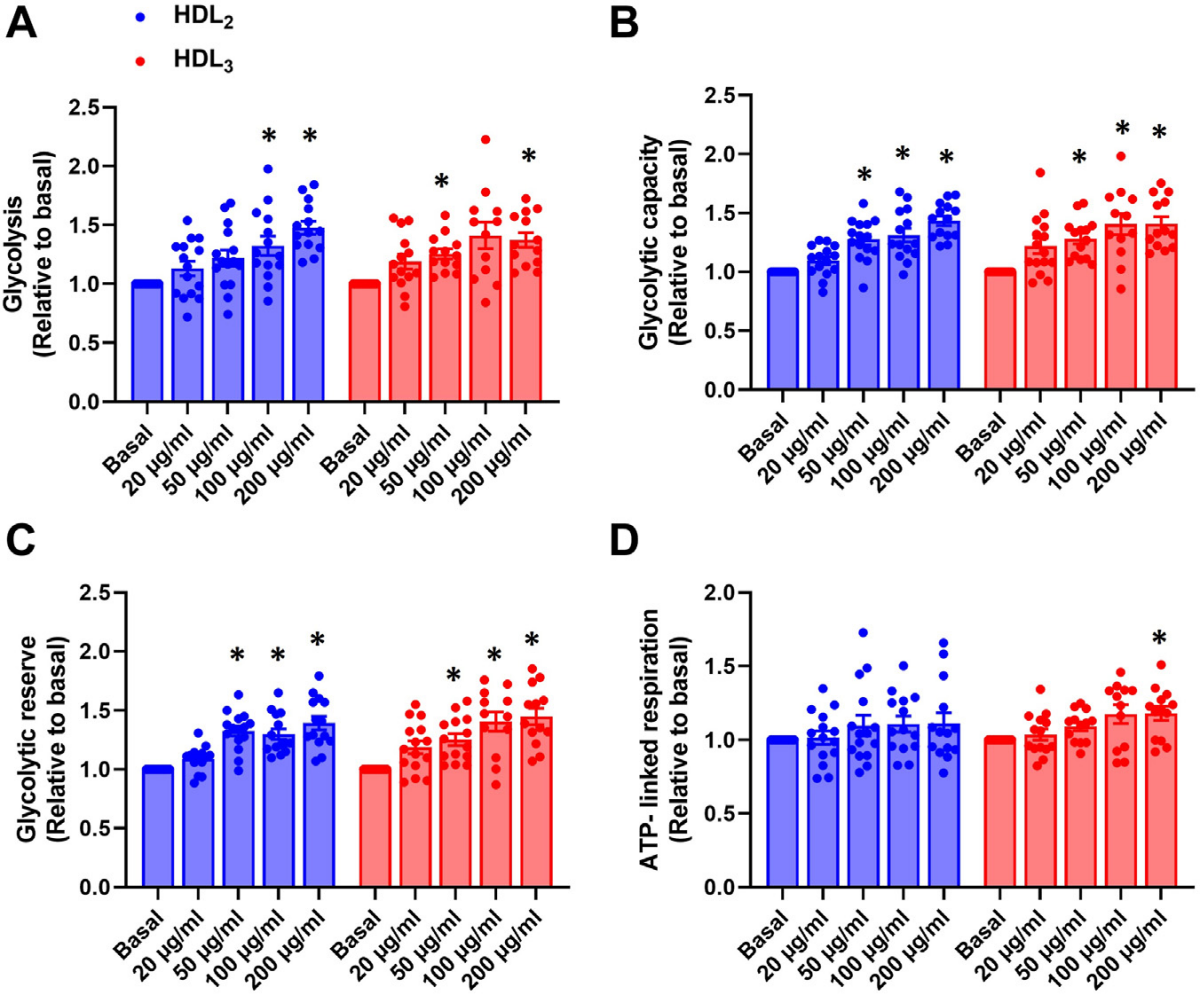
Effects of human HDL_2_ and HDL_3_ on ECAR and OCR of intact mouse myotubes after high glucose conditioning. A: Glycolysis, (B) glycolytic capacity, (C) glycolytic reserve, and (D) ATP-linked respiration after 4 h treatment with or without human HDL_2_ or HDL_3_ present (12.5, 25, 50, 100, or 200 μg/ml) in high glucose (25 mmol/l) conditioning. Results are presented as means ± SEM relative to basal from 12 to 15 individual experiments (*n* = 12 for HDL_3_ 100 μg/ml in each glycolytic flux parameter and ATP-linked respiration; *n* = 13 for HDL_3_ 50 μg/ml in glycolysis and ATP-linked respiration, and HDL_3_ 200 μg/ml in each glycolytic flux parameter; *n* = 14 for HDL_2_ 100 and 200 μg/ml, HDL_3_ 20 μg/ml in ATP-linked respiration, HDL_2_ 100 and 200 μg/ml in each glycolytic parameter, HDL_2_ 20 μg/ml in glycolytic reserve and HDL_3_ 20 μg/ml in glycolysis; *n* = 15 for the basal, HDL_2_ 20 and 50 μg/ml in ATP-linked respiration, HDL_2_ 50 μg/ml in each glycolytic flux parameter, HDL_2_ 20 μg/ml in glycolysis and glycolytic capacity and HDL_3_ 20 μg/ml in glycolytic capacity and glycolytic reserve). ∗Statistically significant versus basal (*P* < 0.05, two-way ANOVA with Tukey’s post hoc comparison). Mean ± SEM of basal values: glycolysis 1.21 ± 0.07 mpH/min/μg; glycolytic capacity 2.38 ± 0.11 mpH/min/μg; glycolytic reserve 1.17 ± 0.05 mpH/min/μg; ATP-linked respiration 3.92 ± 0.13 pmol/min/μg. ECAR, extracellular acidification rate; OCR, oxygen consumption rate.

The effects of HDL_2_ and HDL_3_ on ECAR and OCR during high glucose (25 mmol/l) conditioning were measured with a Seahorse XFe96 analyzer. Several glycolytic flux parameters (glycolysis, glycolytic capacity, and glycolytic reserve) and cellular respiration parameters (basal respiration, proton leak, maximal respiration, ATP-linked respiration, and spare capacity) were analyzed from the data. Furthermore, coupling-control efficiencies (R-L control efficiency ((R-L)/R), E-L coupling control ((E-L)/E) and E-R control efficiency ((E-R)/E)) and FCRs (L/R coupling-control ratio, L/E coupling-control ratio and R/E control ratio) were analyzed from the data. The three highest concentrations (50, 100, and 200 μg/ml) of HDL_2_ and HDL_3_ increased glycolysis, glycolytic capacity, and glycolytic reserve (Fig. 1A–C). Only the highest concentration of HDL_3_ (200 μg/ml) significantly increased ATP-linked respiration but the difference was not significant between HDL_2_ and HDL_3_ (Fig. 1D and supplemental Table S2). Both HDL subclasses with the highest concentration (200 μg/ml) increased R-L control efficiency, which was also seen as a decreased L/R coupling-control ratio (supplemental Fig. S1). These illustrate the increased fraction of basal respiration coupled to ATP phosphorylation. There were no changes in other FCRs or coupling-control efficiencies (supplemental Table S3). The HDL subclasses had a similar response on glucose metabolism as there were no significant differences in the responses between HDL subclasses (Fig. 1 and supplemental Fig. S1).

#### Effects of HDL_2_ and HDL_3_ on cellular respiration and glycolytic flux parameters upon oleic acid conditioning in cultured mouse myotubes

**Fig. 2:**
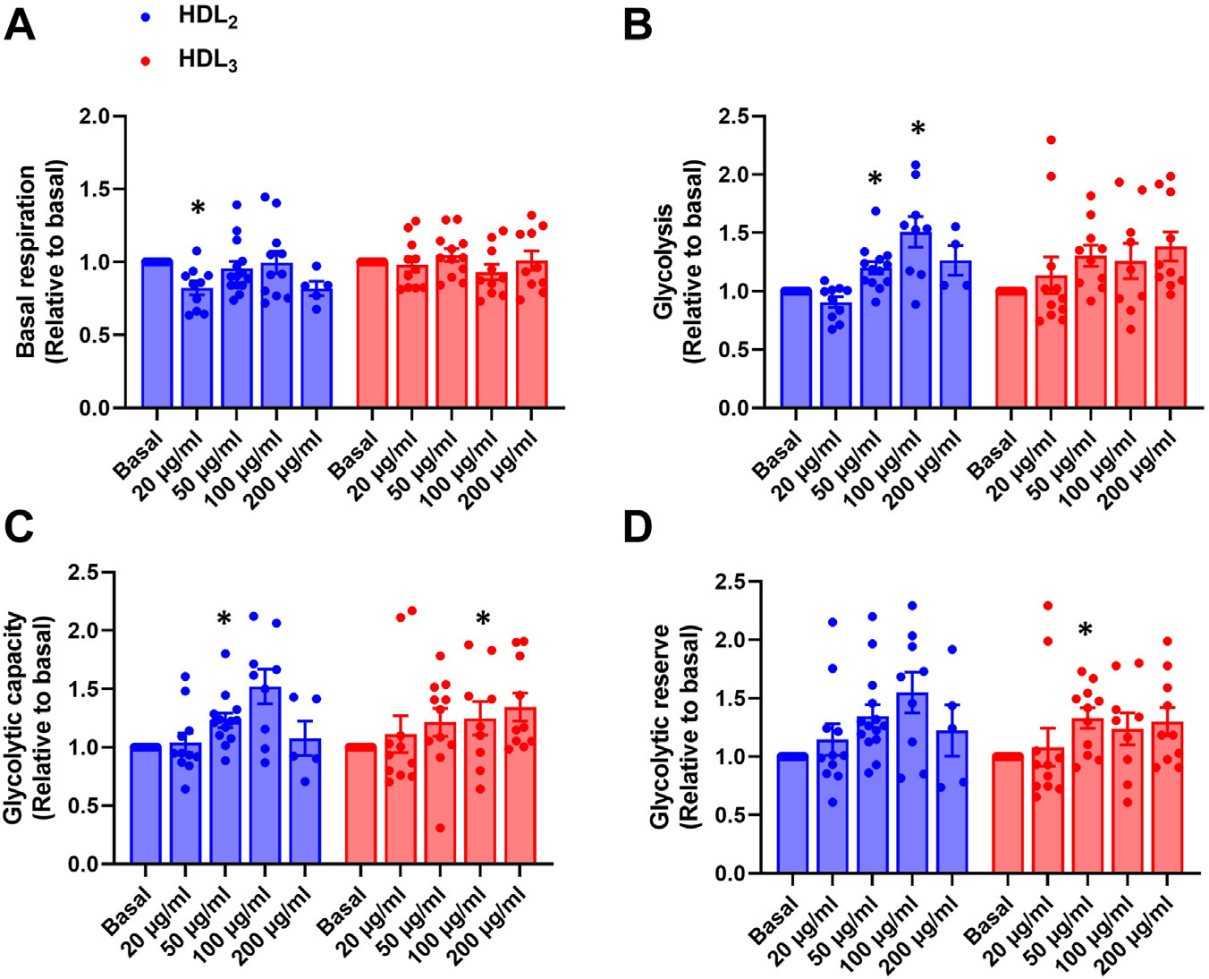
Effects of human HDL_2_ and HDL_3_ on OCR and ECAR of intact mouse myotubes after oleic acid conditioning. A: Basal respiration, (B) glycolysis, (C) glycolytic capacity, and (D) glycolytic reserve after 4 h treatment with or without human HDL_2_ or HDL_3_ present (12.5, 25, 50, 100, or 200 μg/ml) in oleic acid (100 μmol/l albumin-bound oleic acid with 5.5 mmol/l glucose) conditioning. Results are presented as means ± SEM relative to basal from 4 to 14 individual experiments (*n* = 4 for HDL_2_ 200 μg/ml in glycolysis; *n* = 5 for HDL_2_ 200 μg/ml in basal respiration, glycolytic capacity and glycolytic reserve; *n* = 9 for HDL_2_ 100 μg/ml and HDL_3_ 100 μg/ml in each glycolytic flux parameter; *n* = 10 for HDL_2_ 20 μg/ml, HDL_3_ 100 and 200 μg/ml in basal respiration, HDL_3_ 200 μg/ml in each glycolytic flux parameter and for HDL_2_ 20 μg/ml in glycolysis; *n* = 11 for basal in basal respiration, HDL_2_ 100 μg/ml and HDL_3_ 20 μg/ml in basal respiration, HDL_3_ 20 μg/ml in each glycolytic flux parameter, HDL_2_ 20 μg/ml and HDL_3_ 50 μg/ml in each glycolytic flux parameter; *n* = 12 for basal in each glycolytic flux parameter and HDL_3_ 50 μg/ml in basal respiration; *n* = 13 for HDL_2_ 50 μg/ml in glycolysis and glycolytic capacity; *n* = 14 for HDL_2_ 50 μg/ml in basal respiration and glycolytic reserve). ∗Statistically significant versus basal (*P* < 0.05, two-way ANOVA with Tukey’s post hoc comparison). Mean ± SEM of basal values: basal respiration 3.93 ± 0.20 pmol/min/μg; glycolysis 0.76 ± 0.05 mpH/min/μg; glycolytic capacity 1.31 ± 0.10; glycolytic reserve 0.55 ± 0.06. ECAR, extracellular acidification rate; OCR, oxygen consumption rate.

The effects of HDL_2_ and HDL_3_ on OCR and ECAR were also measured during oleic acid conditioning (100 μmol/l albumin-bound oleic acid with low glucose (5.5 mmol/l)) in the same way as described above. The lowest concentration of HDL_2_ reduced basal respiration but the difference was not significant between HDL_2_ and HDL_3_ (Fig. 2A and supplemental Table S4). R-L control efficiency was decreased by 100 μg/ml HDL_3_, which was also seen as an increase in L/R coupling-control ratio (supplemental Fig. S2A, B), indicating a decreased proportion of basal respiration coupled to ATP production. Additionally, E-R control efficiency, which illustrates the reserve capacity of mitochondrial respiration, was increased by 50 and 100 μg/ml HDL_2_, and this was shown as a decrease in R/E control ratio caused by 100 μg/ml of both HDL subclasses (supplemental Fig. S2C, D and supplemental Table S5). Glycolysis was increased by 50 and 100 μg/ml HDL_2_ and glycolytic reserve was elevated by 50 μg/ml HDL_3_ (Fig. 2B, D). Glycolytic capacity was elevated by both HDL subclasses at a concentration of 100 μg/ml (Fig. 2C). Again, the HDL subclasses had a similar response on fatty acid metabolism as there were no significant differences in the responses between HDL subclasses (Fig. 2 and supplemental Fig. S2).

#### Effects of HDL_2_ and HDL_3_ on glucose oxidative metabolism and FAO in permeabilized mouse myotubes

**Fig. 3:**
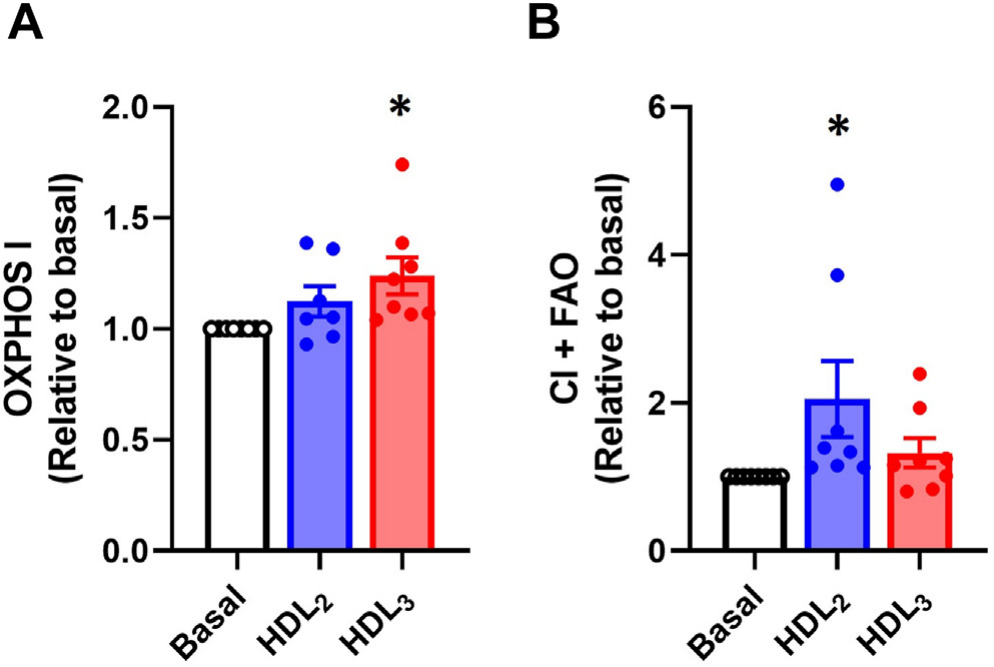
Effects of human HDL_2_ and HDL_3_ on glucose oxidative metabolism and fatty acid oxidation in permeabilized mouse myotubes. A: OXPHOS I with pyruvate (5 mmol/l), malate (2 mmol/l) and ADP + Mg^2+^ (5 mmol/l + 3 mmol/l), and (B) CI + FAO with malate (0.1 mmol/l), ADP + Mg^2+^ (5 mmol/l + 3 mmol/l) and palmitoylcarnitine (10 μmol/l) in permeabilized mouse myotubes after 4 h treatment with or without human HDL_2_ or HDL_3_ present (100 μg/ml). Mitochondrial respiration was normalized to citrate synthase activity (O_2_ pmol/((s x ml)/(U/ml)). Results are presented as means ± SEM relative to basal from 7 to 8 individual experiments (*n* = 7 for HDL_2_ in OXPHOS I, *n* = 8 for basal, CI + FAO and HDL_3_ in OXPHOS I). ∗Statistically significant versus basal (*P* < 0.05, one-way ANOVA with Tukey’s post hoc comparison and Kruskal–Wallis test with Dunn’s multiple comparisons test). Mean ± SEM of basal values: OXPHOS I 2932 ± 140 O_2_ pmol/((s x ml)/(U/ml)); CI + FAO 994 ± 52 O_2_ pmol/((s x ml)/(U/ml)). FAO, fatty acid oxidation.

To obtain further insight into mitochondrial respiratory complex functions, we also studied whether HDL_2_ and HDL_3_ affected glucose oxidative metabolism (without glycolysis) and fatty acid (palmitoylcarnitine) oxidation in permeabilized myotubes by using high-resolution respirometry. Based on the results with intact mouse myotubes, 100 μg/ml of HDL subclasses were used. HDL_3_ increased respiratory complex I-mediated mitochondrial respiration compared to basal upon pyruvate conditioning (Fig. 3A). HDL_2_ had a similar effect upon fatty acid conditioning as respiratory complex I-mediated FAO (CI + FAO) was elevated (Fig. 3B). However, there were no significant differences between HDL_2_ and HDL_3_ responses (Fig. 3). HDL subclasses did not have an effect on other mitochondrial respiration states, coupling control efficiencies or flux control ratios (supplemental Tables S6–S8).

#### LDL controls in mouse myotube experiments

In addition to lipoprotein-free medium used as a control, LDL (100 μg/ml) was included as a lipoprotein control in ECAR and OCR measurements. In high glucose treatment, only proton leak was increased by LDL treatment when compared to basal (medium control) (supplemental Fig. S3A). There were no significant differences between medium control and LDL responses during fatty acid treatment (supplemental Fig. S4).

### Effects of HDL subclasses on energy metabolism in human skeletal muscle cells

We went further with the energy substrate metabolism measurements by studying the effects of human HDL_2_ and HDL_3_ on glucose and fatty acid uptake and oxidation in human primary myotubes. Again, we used a concentration gradient from 12.5 to 200 μg/ml (as total HDL protein) of HDL subclasses to establish proper concentrations for further experiments.

#### Effects of HDL_2_ and HDL_3_ on glucose metabolism in cultured human myotubes

**Fig. 4:**
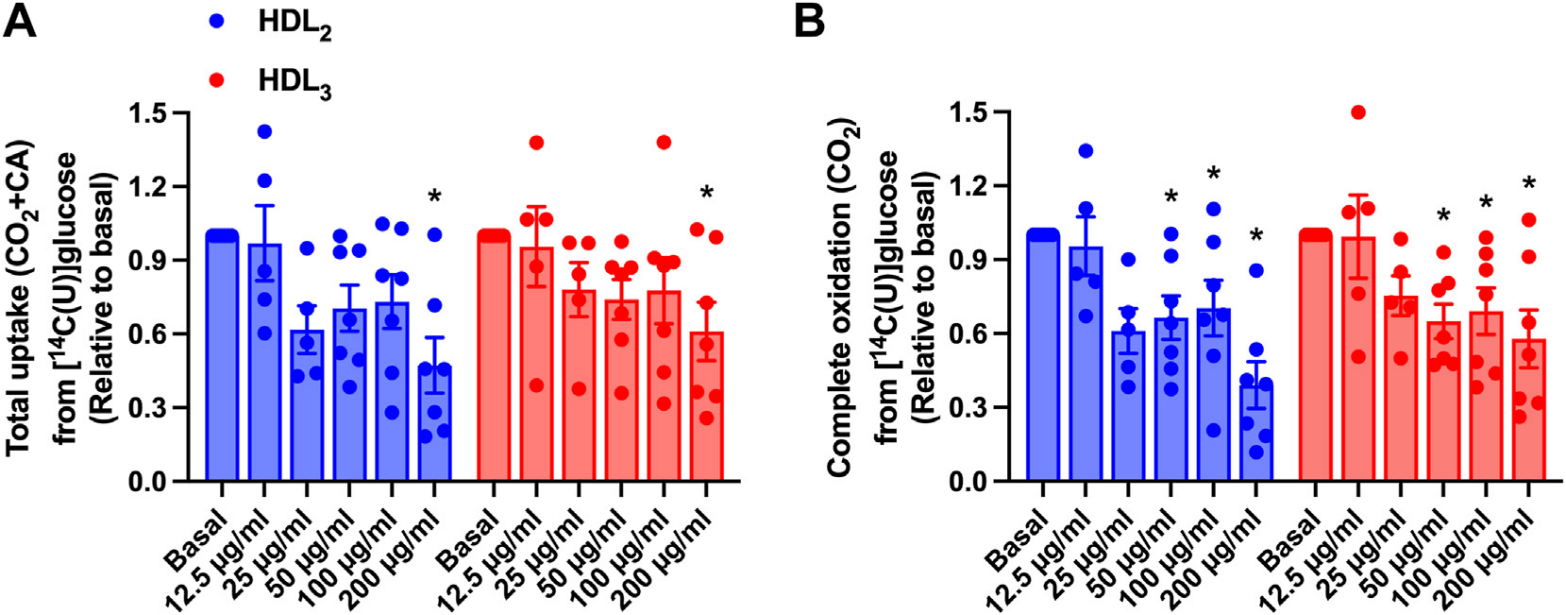
Effect of human HDL_2_ and HDL_3_ on glucose metabolism in human myotubes. A: Total uptake (CO_2_ + CA) and (B) complete oxidation (CO_2_) of 200 μmol/l [^14^C] glucose in cultured human myotubes after 4 h treatment with or without human HDL_2_ or HDL_3_ present (12.5, 25, 50, 100, or 200 μg/ml). Results are presented as means ± SEM relative to basal from 5 to 7 individual experiments (*n* = 5 for the 12.5 and 25 μg/ml concentrations, whereas *n* = 7 for the basal and 50, 100, and 200 μg/ml concentrations). ∗Statistically significant versus basal (*P* < 0.05, two-way ANOVA with Tukey’s post hoc comparison). Mean ± SEM of basal values: total glucose uptake 74.0 ± 19.3 nmol/mg protein; complete glucose oxidation 41.8 ± 9.2 nmol/mg protein. CA, cell-associated radioactivity.

Both human HDL_2_ and HDL_3_ decreased total glucose uptake (CO_2_+CA) and complete oxidation (CO_2_ formation) of glucose in cultured human myotubes (Fig. 4). Only the highest concentration used (200 μg/ml) significantly decreased the total cellular uptake, whereas complete oxidation was decreased by the three highest concentrations (50, 100, and 200 μg/ml). The HDL subclasses had a similar response on glucose metabolism (Fig. 4).

#### Effects of HDL_2_ and HDL_3_ on fatty acid metabolism in cultured human myotubes

**Fig. 5:**
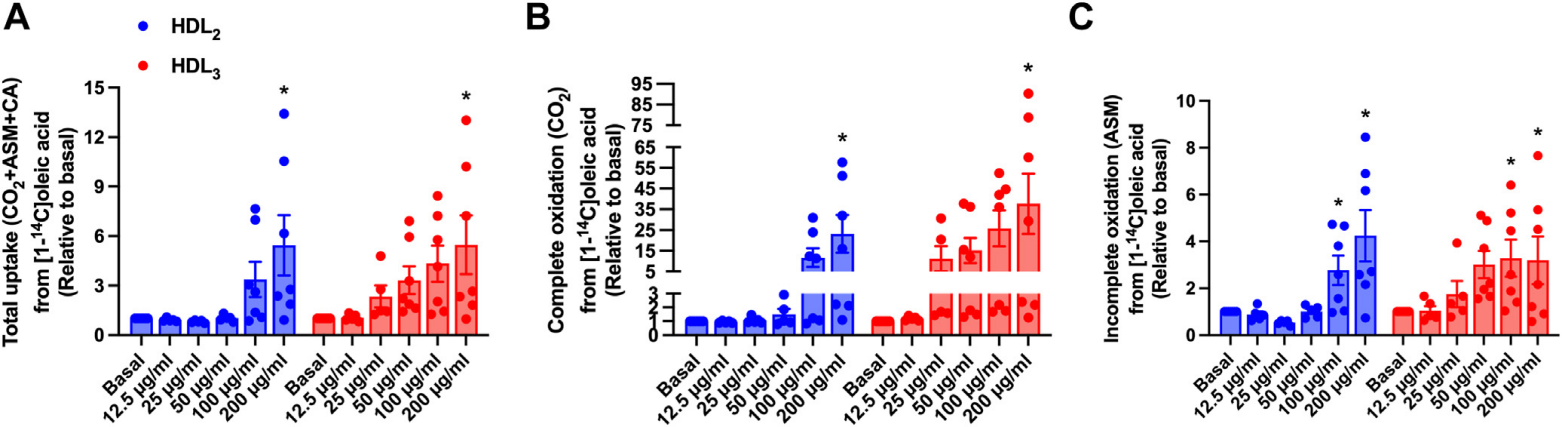
Effect of human HDL_2_ and HDL_3_ on extracellular oleic acid metabolism in human myotubes. A: Total uptake (CO_2_ + ASM + CA), (B) complete oxidation (CO_2_), and (C) incomplete oxidation (ASM) of 100 μmol/l [^14^C]oleic acid in cultured human myotubes after 4 h treatment with or without human HDL_2_ or HDL_3_ present (12.5, 25, 50, 100, or 200 μg/ml). Values are presented as means ± SEM relative to basal from 5 to 7 individual experiments (*n* = 5 for the 12.5 and 25 μg/ml concentrations, whereas *n* = 7 for the basal and 50, 100, and 200 μg/ml concentrations). ∗Statistically significant versus basal (*P* < 0.05, two-way ANOVA with Tukey’s post hoc comparison). Mean ± SEM of basal values: total oleic acid uptake 214.7 ± 31.4 nmol/mg protein; complete oleic acid oxidation 15.4 ± 2.3 nmol/mg protein; incomplete oleic acid oxidation 95.9 ± 22.5 nmol/mg protein; fractional oxidation 0.07 ± 0.01. ASM, acid-soluble metabolite; CA, cell-associated radioactivity.

We also examined the role of HDL subclasses on oleic acid metabolism in the same manner as for glucose. HDL_2_ and HDL_3_ markedly increased total cellular uptake (CO_2_+ASM+CA), complete oxidation (CO_2_), and incomplete oxidation (fatty acid β-oxidation, ASM) of oleic acid (Fig. 5). Only the highest concentration (200 μg/ml) significantly increased the total cellular uptake, whereas oxidation (complete and incomplete) was increased by both of the two highest concentrations (100 and 200 μg/ml). The HDL subclasses had a similar response on oleic acid metabolism (Fig. 5). Based on the results obtained from glucose and fatty acid metabolism, we decided to use the concentration of 100 μg/ml HDL_2_ or HDL_3_ for further experiments in human skeletal muscle cells.

Because the effects of HDL_2_ and HDL_3_ on oleic acid metabolism were so striking, we wanted to examine whether the obtained results were caused by changes in cellular substrate fluxes, since the data described in Figs. 4 and 5 were from an acute 4 h incubation of the cells. Hence, we wanted to see whether pretreatment of the cells with unlabeled oleic acid for 24 h would produce similar effects and responses as the acute treatment. The effects by HDL_2_ and HDL_3_ on oleic acid metabolism were similar after both acute treatment and a 24 h pretreatment (supplemental Fig. S5). Thus, the effects by HDL_2_ and HDL_3_ on oleic acid metabolism were due to functional metabolic changes.

#### Effect of HDL on oleic acid distribution into cellular lipids in cultured human myotubes

**Fig. 6:**
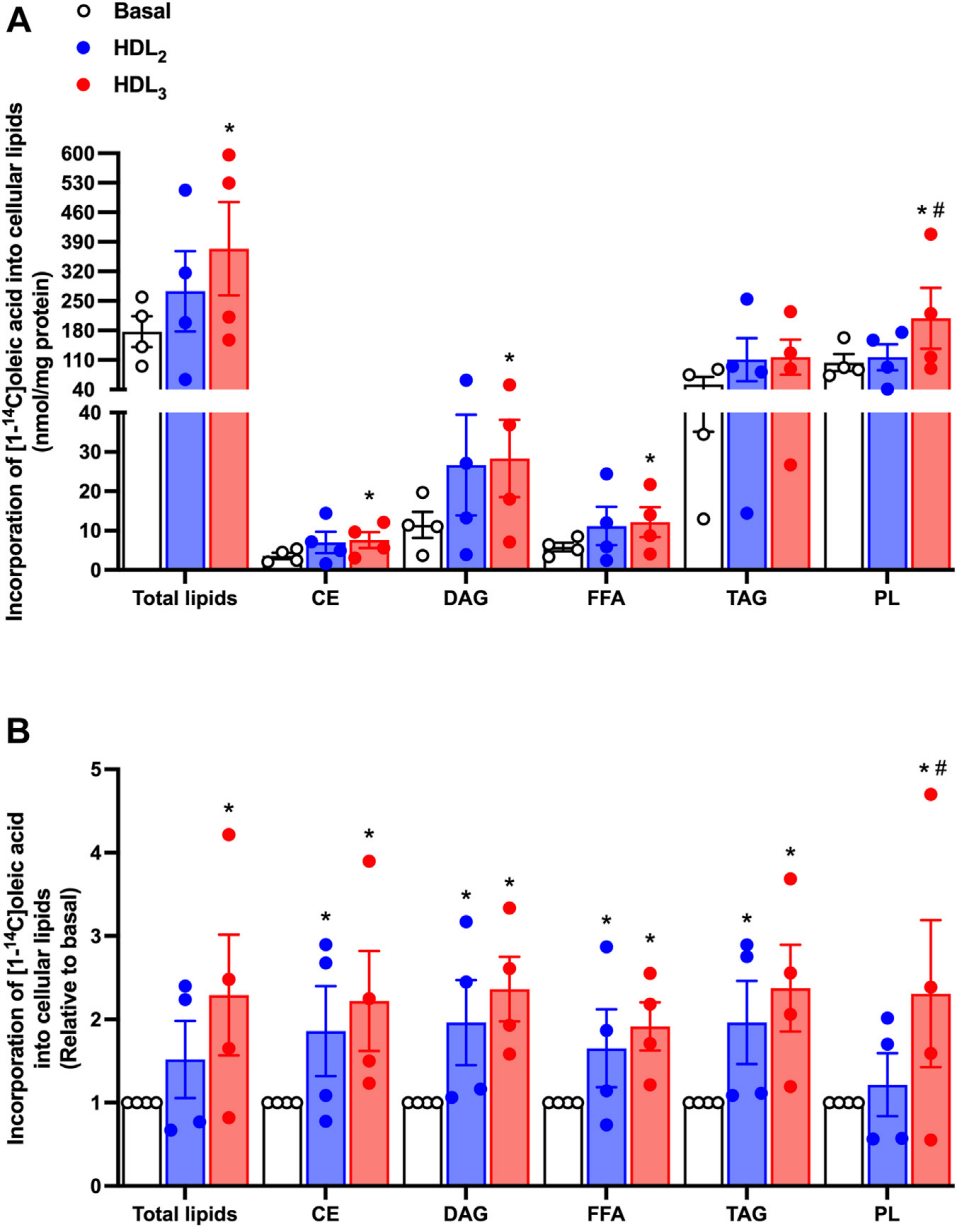
Lipid distribution. After 8 h accumulation of [^14^C]oleic acid with or without 100 μg/ml of human HDL_2_ or HDL_3_ present, the distribution of oleic acid into lipid classes was measured. Values are presented as means ± SEM from four individual experiments (n = 4) in (A) absolute values or (B) relative to basal. ∗Statistically significant versus basal (*P* ≤ 0.05, SPSS linear mixed model analysis). ^#^Statistically significant versus HDL_2_ (*P* < 0.05, SPSS linear mixed model analysis). CE, cholesteryl ester; DAG, diacylglycerol; FFA, free fatty acids; PL, phospholipids; TAG, triacylglycerol.

We next investigated how HDL_2_ and HDL_3_ affected the distribution of oleic acid into cellular lipids as part of cellular lipid species to get more insights in cellular fatty acid pathways (Fig. 6). A slightly longer incubation time was used (8 h) to ensure a measurable response. The incorporation of oleic acid into all lipid classes except TAG was increased by HDL_3_ when expressed in absolute values (Fig. 6A), as well as the total lipid incorporation. When presented relative to basal, we observed that both HDL_2_ and HDL_3_ treatment increased the incorporation of oleic acid into CE, diacylglycerol, and TAG, as well as increased the FFAs pool, whereas HDL_3_ but not HDL_2_ increased incorporation into PL as well as total lipid incorporation of oleic acid (Fig. 6B). Furthermore, HDL_3_ increased the incorporation of oleic acid into PL more when compared to the outcome by HDL_2_ (Fig. 6).

#### Effects of HDL_2_ and HDL_3_ on mRNA expression of selected genes in cultured human myotubes

**Fig. 7:**
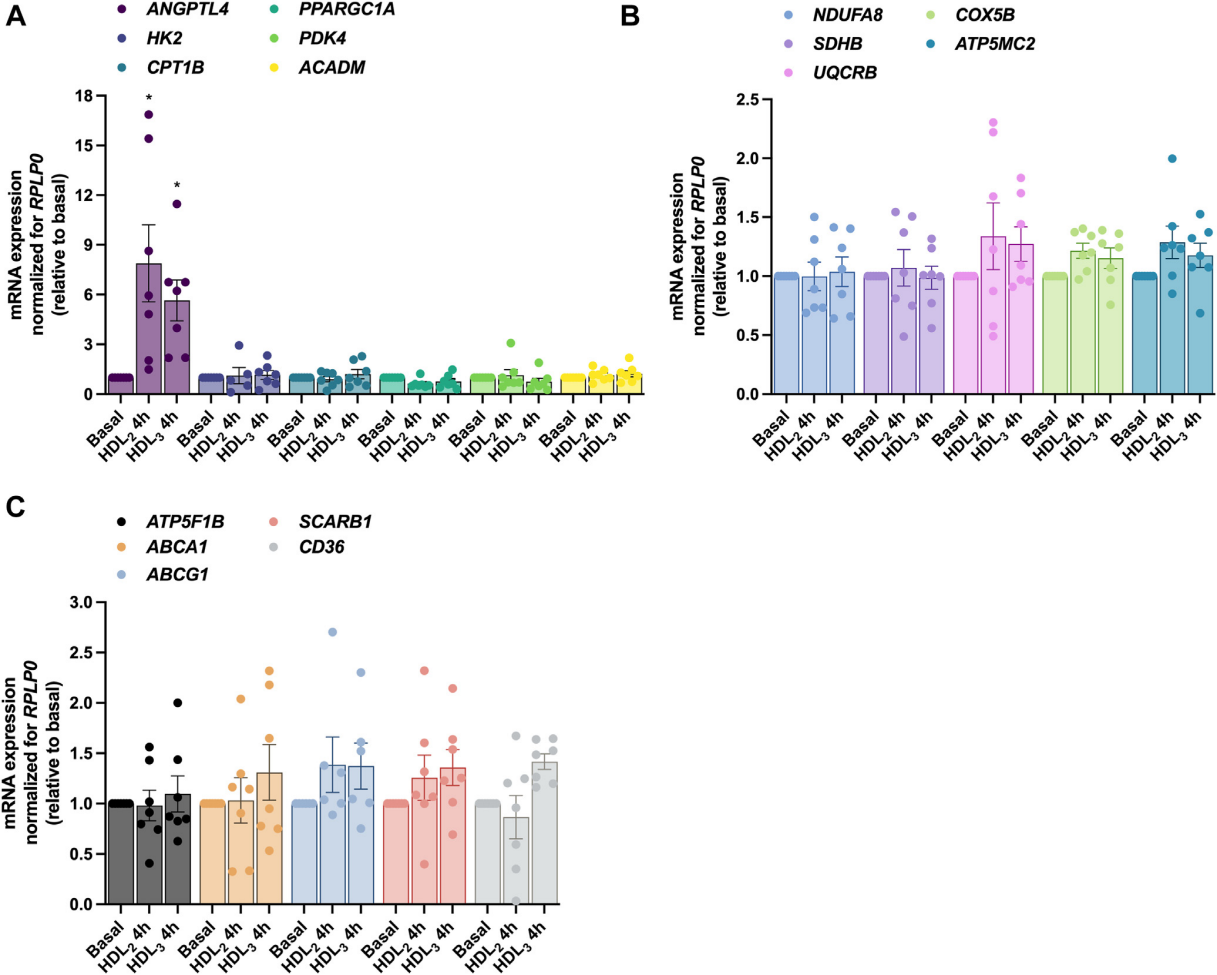
Effect of 4 h treatment with human HDL_2_ and HDL_3_ on mRNA expressions of human myotubes. Total RNA was isolated, and mRNA reversely transcribed before expressions of a selection of genes were assessed by quantitative real-time PCR. Values are presented as means ± SEM (*n* = 7 in each group) and corrected for the average of the housekeeping gene *RPLP0*. The results were normalized to the results of the mRNA expression for myotubes from basal samples. A: mRNA expression of selected genes important in glucose and fatty acid metabolism. B: mRNA expression of selected genes in the electron transport chain. C: mRNA expression of HDL transporters. ∗Statistically significant versus basal (*P* < 0.05, two-way ANOVA). *ABCA1*, ATP-binding cassette subfamily A member 1; *ABCG1*, ATP-binding cassette subfamily G member 1; *ACADM*, acyl-CoA dehydrogenase medium chain; *ANGPTL4*, angiopoietin like 4; *ATP5F1B*, ATP synthase F1 subunit beta; *ATP5MC2*, ATP synthase membrane subunit c locus 2; *CD36*, CD36 molecule; *COX5B*, cytochrome c oxidase subunit 5B; *CPT1B*, carnitine palmitoyltransferase 1B; *HK2*, hexokinase 2; *NDUFA8*, NADH:ubiquinone oxidoreductase subunit A8; *PDK4*, pyruvate dehydrogenase kinase 4; *PPARGC1A*, peroxisome proliferator–activated receptor gamma coactivator 1 alpha; RPLP0, ribosomal protein lateral stalk subunit P0; *SCARB1*, scavenger receptor class B member 1; *SDHB*, succinate dehydrogenase complex iron sulfur subunit B; *UQCRB*, ubiquinol-cytochrome c reductase binding protein.

**Fig. 8:**
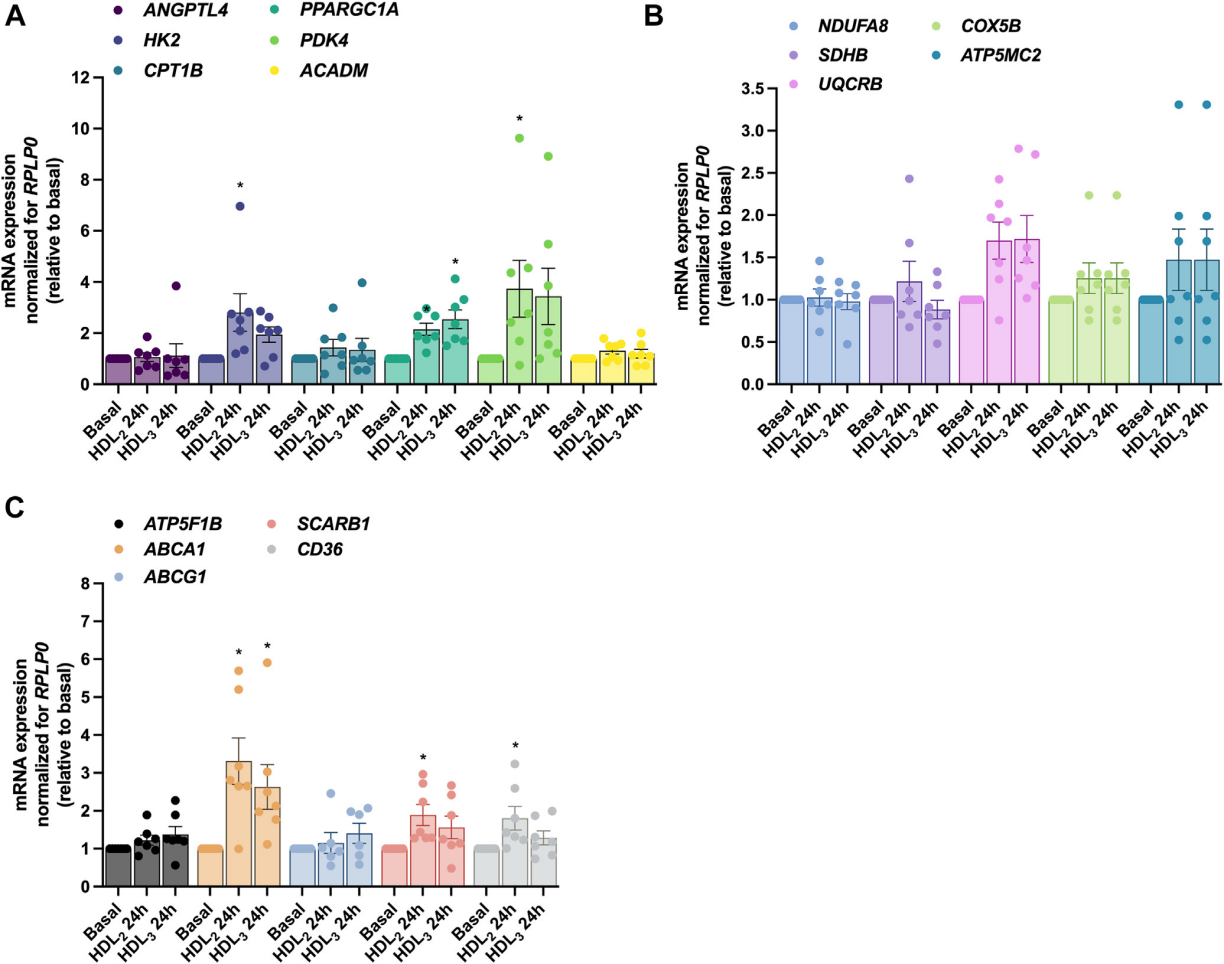
Effect of 24 h treatment with human HDL_2_ and HDL_3_ on mRNA expressions of human myotubes. Total RNA was isolated, and mRNA reversely transcribed before expressions of a selection of genes were assessed by quantitative real-time PCR. Values are presented as means ± SEM (*n* = 7 in each group) and corrected for the average of the housekeeping gene *RPLP0*. The results were normalized to the results of the mRNA expression for myotubes from basal samples. A: mRNA expression of selected genes important in glucose and fatty acid metabolism. B: mRNA expression of selected genes in the electron transport chain. C: mRNA expression of HDL transporters. ∗Statistically significant versus basal (*P* < 0.05, two-way ANOVA). *ABCA1*, ATP-binding cassette subfamily A member 1; *ABCG1*, ATP-binding cassette subfamily G member 1; *ACADM*, acyl-CoA dehydrogenase medium chain; *ANGPTL4*, angiopoietin like 4; *ATP5F1B*, ATP synthase F1 subunit beta; *ATP5MC2*, ATP synthase membrane subunit c locus 2; *CD36*, CD36 molecule; *COX5B*, cytochrome c oxidase subunit 5B; *CPT1B*, carnitine palmitoyltransferase 1B; *HK2*, hexokinase 2; *NDUFA8*, NADH:ubiquinone oxidoreductase subunit A8; *PDK4*, pyruvate dehydrogenase kinase 4; *PPARGC1A*, peroxisome proliferator activated receptor gamma coactivator 1 alpha; RPLP0, ribosomal protein lateral stalk subunit P0; *SCARB1*, scavenger receptor class B member 1; *SDHB*, succinate dehydrogenase complex iron sulfur subunit B; *UQCRB*, ubiquinol-cytochrome c reductase binding protein.

To examine the background of functional changes observed in human myotubes, we continued by studying the mRNA expression of selected genes relevant for glucose and fatty acid metabolism and for the HDL receptors (Figs. 7 and 8). As a strong response to the HDL subclasses were observed on glucose and oleic acid metabolism already after a 4 h (acute) treatment, we also wanted to investigate if this short incubation time could induce possible differences in mRNA expressions (Fig. 7). But since 4 h may be a short time for changes in mRNA expression, we further analyzed the expression of the same genes after a 24 h treatment with HDL_2_ and HDL_3_ (Fig. 8). Acute treatment with HDL_2_ and HDL_3_ markedly increased mRNA expression of angiopoietin like 4 (*ANGPTL4*), whereas the expression of other genes was not affected (Fig. 7A–C). *ANGPTL4* expression seemed to be an acute, transient increase as mRNA expression attenuated to basal levels again with a 24 h pretreatment (Fig. 8A). On the other hand, a 24 h pretreatment with HDL_2_ and HDL_3_ increased the mRNA expressions of peroxisome proliferator–activated receptor gamma coactivator 1 alpha (*PPARGC1A*) (Fig. 8A) and *ABCA1* (Fig. 8C). Pretreatment of 24 h with HDL_2_, but not HDL_3_, increased mRNA expression of hexokinase 2 (*HK2*) and pyruvate dehydrogenase kinase 4 (*PDK4*) (Fig. 8A), scavenger receptor class B member 1 (*SCARB1*), and *CD36* (Fig. 8C). There were no changes in the mRNA expression levels of genes related to the electron transport chain (Fig. 8B).

#### LDL controls in human myotube experiments

In addition to the lipoprotein-free medium used as a control, LDL (100 μg/ml) was included as a lipoprotein control. No effects by LDL were observed on glucose or oleic acid metabolism in cultured human myotubes (supplemental Fig. S6).

#### Effects of d-rHDL and apoA-I on glucose and fatty acid metabolism

**Fig. 9:**
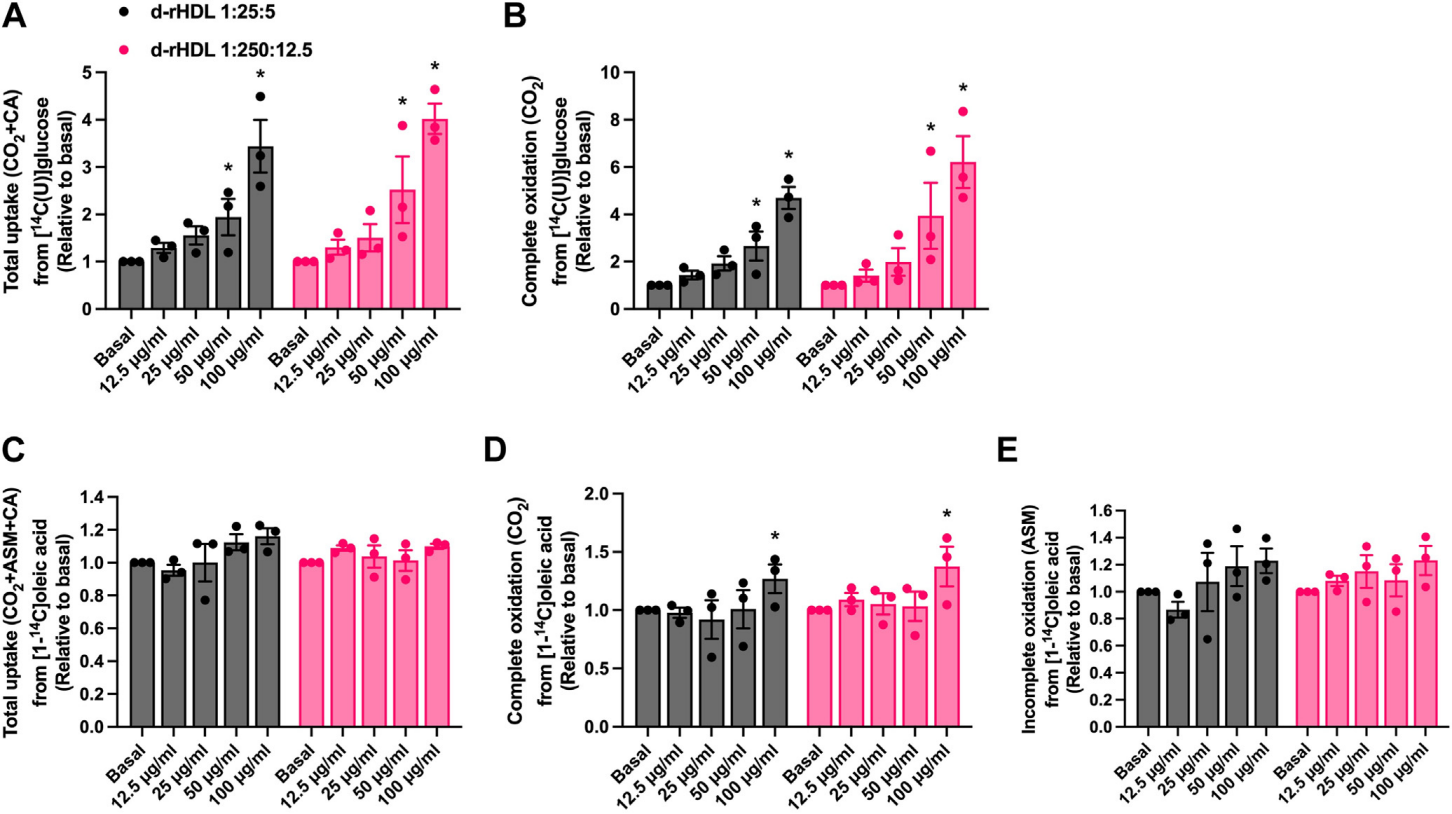
Effect of discoidal reconstituted HDLs (d-rHDLs) on glucose and oleic acid metabolism. A: Total uptake (CO_2_ + CA) and (B) complete oxidation (CO_2_) of 200 μmol/l [^14^C]glucose, and (C) total uptake (CO_2_ + ASM + CA), (D) complete oxidation (CO_2_) and (E) incomplete oxidation (ASM) of 100 μmol/l [^14^C]oleic acid in cultured human myotubes after 4 h incubation with or without d-rHDL 1:25:5 (apoA-I:PL:C) or d-rHDL 1:250:12.5 (apoA-I:PL:C) present (12.5, 25, 50, or 100 μg/ml). Values are presented as means ± SEM from 3 individual experiments (n = 3). ∗Statistically significant versus basal (*P* < 0.05, one-way ANOVA with Dunnett’s correction). Mean ± SEM of basal values: Total glucose uptake 24.1 ± 3.8 nmol/mg protein; complete glucose oxidation 12.2 ± 2.3 nmol/mg protein; total oleic acid uptake 147.7 ± 0.6 nmol/mg protein; complete oleic acid oxidation 8.2 ± 1.0 nmol/mg protein; incomplete oleic acid oxidation 39.9 ± 3.1 nmol/mg protein. ASM, acid-soluble metabolites; CA, cell-associated radioactivity; d-rHDL, discoidal, reconstituted HDL.

Additionally, we examined the acute effects of d-rHDL and apoA-I on glucose and fatty acid metabolism. Human myotubes were incubated for 4 h with different concentrations of two prepared d-rHDLs with molar ratios 1:25:5 and 1:250:12.5 of apoA-I:PC:C. The two highest concentrations (50 and 100 μg/ml) increased total cellular uptake and complete oxidation of glucose after incubation of both d-rHDLs (Fig. 9A, B). Only the highest concentration (100 μg/ml) of d-rHDLs increased the complete oxidation of oleic acid (Fig. 9D) whereas total cellular uptake and incomplete oxidation was unaffected by d-rHDLs (Fig. 9C, E). Lipid-free apoA-I did not have much response on either glucose or oleic acid metabolism (supplemental Fig. S7); only the 25 μg/ml concentration increased total cellular uptake of glucose by approximately 20% (supplemental Fig. S7A).

## Discussion

The aim of this study was to investigate the acute effects of human HDL subclasses, HDL_2_ and HDL_3_, on glucose and fatty acid metabolism in mouse and human skeletal muscle myotubes and whether HDL subclasses have distinct metabolic regulating roles. Our results demonstrated that in mouse myotubes HDL increased glycolytic parameters that led to more efficient ATP production observed in intact myotubes when glucose was the main energy substrate. However, there was only a slight impact of HDL on FAO in mouse myotubes. Unlike mouse myotubes, human myotubes responded to HDL treatment by decreasing glucose uptake and oxidation when glucose acted as an energy substrate and increasing fatty acid uptake and oxidation when oleic acid was provided as the main energy substrate. This was in line with increased mRNA expression of *ANGPTL4*, an enhancer of lipid metabolism, in the early phase of HDL treatment of human myofibers. Between HDL subclasses, HDL_2_ and HDL_3,_ there were no major differences on regulation of myotube energy metabolism on mouse or human myotubes. To our knowledge, this is the first study to examine if HDL subclasses differ in their effect on skeletal muscle cell energy metabolism and how HDL is modulating the energy metabolism of skeletal muscle cells from two different species, mouse and human.

At the systemic level, HDL and its main protein component apoA-I have been identified as one of the factors affecting glucose homeostasis (2, 3, 12). Lipid free apoA-I increases glucose uptake in mouse skeletal muscle and has a role in regulating insulin sensitivity in subjects with impaired glucose tolerance (2, 12, 28). However, results based on in vitro measurements are controversial. ApoA-I has been shown to activate the insulin receptor Akt pathway, leading to increased glucose disposal in primary human skeletal muscle cells, but fails to enhance glucose uptake in isolated mouse skeletal muscle tissue (3, 28). Our results with mouse myotubes support a promoting role of HDL on glucose metabolism as glycolytic flux parameters were increased in intact myotubes after treatment with both HDL subclasses, suggesting that HDL_2_ and HDL_3_ markedly promote glucose utilization through the glycolytic pathway. Moreover, a slight increase in ATP-linked cellular respiration by HDL_3_, also seen as an increase in R-L control efficiency and decreased R/L coupling-control ratio caused by both HDL subclasses, suggests improved efficiency for basal respiration coupled to ATP phosphorylation. Permeabilized mouse myotubes had a similar response, as complex I mediated mitochondrial respiration was increased by HDL_3_ but not after HDL_2_ treatment. Interestingly, it has been shown that physical exercising but also fasting, have enhancing effect on mitochondrial integrity and respiration efficiency (29, 30). Further investigations are needed to find if the difference in apoA-I amount between HDL_2_ and HDL_3_ particles (31) leads HDL_3_ to have a stronger role in enhancing glucose metabolism, coupling efficiency and complex I mediated OXPHOS.

In contrast to mouse myotube results, the response seems opposite in human myotubes as both HDL_2_ and HDL_3_ markedly decreased the uptake of glucose and its oxidation. On the gene expression level, *HK2*, which catalyzes the first step in glucose metabolism by directing glucose into glycolysis (32, 33), was upregulated after 24 h treatment with HDL_2_. It might suggest enhanced glycolytic function, but we did not measure the proportion of glycolysis in human myotubes, and this needs further investigation. On the contrary to HDL_2_ and HDL_3_, nascent d-rHDL increased the uptake and oxidation of glucose. Similar responses have been observed, as rHDL has been shown to activate glucose disposal on the whole body level but also trigger glucose uptake and translocation of the skeletal muscle glucose transporter isoform GLUT4, from cytosolic vesicles to the cell surface in rat skeletal muscle myotubes (12, 34). Differences in the responses between mature, spherical HDL_2_ and HDL_3_ particles and d-rHDL might be explained by differences in HDL particle size and shape or the protein, lipid, and cargo content of HDL particles, which could affect the activation or inhibition of pathways leading to glucose uptake, such as GLUT4 translocation. Furthermore, in our experiments lipid-free apoA-I did not have major effect on the energy metabolism of human myotubes, perhaps pointing to the role of other components in the HDL particle. However, apoA-I has been shown to induce insulin-dependent and independent glucose uptake in human skeletal muscle cells (3), and these differences in the responses need to be clarified. Taken together, the effect of HDL subclasses on glucose metabolism may vary between species but is also sensitive to the composition of HDL particles in a way that needs to be further elucidated.

At the cellular level, human HDL and apoA-I have been shown to acutely increase palmitate oxidation in human myotubes and after 24 h in murine myotubes (12, 35). Our results demonstrated that in intact mouse myotubes, cellular respiration parameters did not show any increase, but glycolytic flux parameters were elevated by both HDL subclasses upon oleic acid conditioning, but not as heavily as after high glucose conditioning, which could be explained due to lower glucose concentration. This is again indicating that both HDL subclasses have the capability to improve anaerobic metabolism also at lower glucose levels (fasted state). However, in permeabilized mouse myotubes, HDL_2_ increased complex I mediated mitochondrial respiration. It has been shown that individuals with a high aerobic fitness level have more larger HDL_2_ particles (9) but also improved maximal capacity for FAO (36, 37), which might indicate a stronger role and connection between HDL_2_ and improved fatty acid utilization.

Our results with human primary myotubes show that already 4 h treatment with either HDL_2_ or HDL_3_ enhanced fatty acid metabolism through increased uptake and oxidation in human myotubes and elevated the expression level of *ANGPTL4*, which is suggested to augment fatty acid uptake and oxidation in skeletal muscle cells through activation of the AMPK pathway during and after exercise (38, 39). Furthermore, both HDL subclasses increased incorporation of oleic acid into cellular lipid species suggesting that HDL enhances the storage of the intracellular lipids in addition to the usage of fatty acids as an energy source. Increased oxidation of oleic acid was also seen after d-rHDL but not after lipid-free apoA-I treatments, which supports the role of HDL as a factor improving fatty acid metabolism but it also might indicate PLs and cholesterol being the major modulators for FAO. However, opposite response has been observed in subjects with T2D as infused rHDL inhibited endogenous adipose lipolysis of TAGs and FAO at whole-body level (13). It might be that T2D induced metabolic differences but also the possible maturation process of rHDL in circulation of T2D subjects could explain distinct results. Combined, these results suggest that HDL subclasses and d-rHDL have an improving effect on FAO, however, the differences in the metabolic profiles might explain the observed variation in the responses between human and mouse.

The observed changes in gene expression after HDL treatment of human skeletal muscle myotubes support the findings that HDL modifies the metabolic behavior of muscle cells. In addition to acutely upregulated *ANGPTL4*, with a 24 h treatment we saw elevated expression of *PPARGC1A*, which is known as a master regulator of mitochondrial biogenesis and function and modeling skeletal muscle fibers towards a more oxidative nature (40), *HK2*, which is needed for glucose phosphorylation and transport into glycolysis, and *PDK4*, which is suggested to function as an energy substrate switch in skeletal muscle cells during fasting and after exercise, leading to a shift from carbohydrate utilization to enhanced FAO (41, 42). These results strongly support the suggested role of HDL in the regulation of skeletal muscle energy metabolism (12, 13, 14, 43). In addition to metabolic genes, we saw that mRNA expression of three HDL receptors increased with a 24 h HDL treatment. Transmembrane receptor *ABCA1*, which was upregulated by both HDL subclasses, has a major role in cholesterol efflux but it is also suggested to improve glucose uptake into mouse muscle cells by mediating GLUT4 translocation and activating insulin signaling via regulation of membrane cholesterol content (44). In addition, HDL_2_ elevated the gene expression of two other HDL-interacting membrane scavenger receptors, *SCARB1*, and *CD36*, known as receptors for cholesterol transport to the liver for degradation (45) and transporter of fatty acids into skeletal muscle cells (45, 46, 47), respectively. CD36 has been shown to increase FAO independently of mitochondrial density, and therefore has a central role in the regulation of skeletal muscle fuel selection (47). An increase in gene expression of these receptors associated with fatty acid metabolism is in line with the results and strengthens the suggestion that HDL subclasses encourage human skeletal muscle cells toward enhanced fatty acid uptake and oxidative metabolism.

Interestingly, our results with mouse and human myofibers differed significantly. Differences related to metabolic pathways and substrate utilization might explain some of the observed variation between mouse and human responses. When glucose is the main substrate, human skeletal muscle myotubes tend to be more glycolytic whereas mouse myotubes rely more on oxidative metabolism (48), which could explain the improvements in glucose oxidation in mouse myotubes but also the decreased oxidation in human myotubes. Oxidation of fatty acids has been shown to function at similar levels in mouse and human in vitro models (48), but this similarity was not clearly visible in our results, which might be a result of different methods and cell culturing conditions. Furthermore, human HDL subclasses were used for this study, even though human and mouse cell models were applied in the measurements. Mouse has normally rather homogenous one HDL class with some differences in the protein content when compared to human HDL (49, 50). Therefore, human HDL subclasses used in this study might have caused some bias in the response when used in the context of mouse myotubes. These differences between human and mouse cell models should be carefully considered when cellular-level studies on HDL function are conducted. Moreover, the detailed protein and lipid composition of HDL subclasses could have added more information about the differences or similarities between subclasses and explained why we did not find a clear difference between subclasses in the responses of cellular metabolism. As we did not analyze the protein and lipid or the cargo content of HDL subclasses in a more detailed way, this is a relevant limitation of the study.

## Conclusions

This study showed that HDL subclasses have a role in modulating the energy metabolism of skeletal muscle cells. In cultured human myotubes, fatty acid uptake and oxidation were increased markedly, and genes related to fatty acid metabolism as well as HDL receptors were upregulated after HDL_2_ and HDL_3_ treatments, but only minor responses were seen in mouse myotubes as complex I mediated OXPHOS was elevated by HDL_2_. In contrast, both HDL_2_ and HDL_3_ decreased glucose uptake and oxidation in human myotubes, whereas both HDL subclasses increased glycolytic function in mouse myotubes, indicating a strong role in glucose disposal through anaerobic metabolic pathway. The different metabolic profiles of human and mouse skeletal muscle cells might explain some differences regarding glucose metabolism. Together these results showed that human HDL subclasses enhance fatty acid utilization and modulate the energy metabolism of human myotubes toward a more oxidative nature, but when glucose is the main energy substrate, HDL_2_ and HDL_3_ enhanced more anaerobic metabolism. In turn, nascent d-rHDL increased glucose oxidative metabolism, which refers to the role of structural and compositional differences between HDL subclasses. Our results suggest that human HDL might affect energy substrate choice, but the role of HDL subclasses needs further detailed investigation. Differences in lipid species, protein content, and cargo content between HDL subclasses might be key physiological factors that we did not examine in this study.

## Data availability

The data is contained within the manuscript and the supplementary files. Nonsignificant data relative to basal values are presented as supplementary figures or tables. The original data and dataset analyzed in the current study are available upon request.

## Supplemental data

This article contains supplemental data.

## Conflict of interest

The authors declare that they have no conflicts of interest with the contents of this article.
