# Supplementary material for: Human HDL subclasses modulate energy metabolism in skeletal muscle cells
Source: J Lipid Res. 2023 Nov 24;65(1):100481. doi: 10.1016/j.jlr.2023.100481 (PMC10770614; doi:10.1016/j.jlr.2023.100481)
Supplement: Supplemental Data [file mmc1.docx]

**Supplemental information**

**Human HDL subclasses modulate energy metabolism in skeletal muscle cells**

Jenny Lund^1^ #, Emilia Lähteenmäki^2^ #, Tiia Eklund^3^, Hege G. Bakke^1^, G. Hege Thoresen^1,4^, Eija Pirinen^5,6,7,8^, Matti Jauhiainen^9^, Arild C. Rustan^1^, Maarit Lehti^2^

1. Section for Pharmacology and Pharmaceutical Biosciences, Department of Pharmacy, University of Oslo, Oslo, Norway

2. Faculty of Sport and Health Sciences, University of Jyväskylä, P.O Box 35, 40014 Jyväskylä, Finland

3. Department of Biological and Environmental Science, University of Jyväskylä, Jyväskylä, Finland

4. Department of Pharmacology, Institute of Clinical Medicine, University of Oslo, Oslo, Norway

5. Research Program for Clinical and Molecular Metabolism, Faculty of Medicine, University of Helsinki, FIN-00290 Helsinki, Finland

6. Research Unit for Biomedicine and Internal Medicine, Faculty of Medicine, University of Oulu, FIN-90220 Oulu, Finland

7. Medical Research Center Oulu, Oulu University Hospital and University of Oulu, Oulu, Finland

8. Biocenter Oulu, University of Oulu, Oulu, Finland

9. Department of Public Health and Welfare, Minerva Foundation Institute for Medical Research and Finnish Institute for Health and Welfare, Helsinki, Finland

**
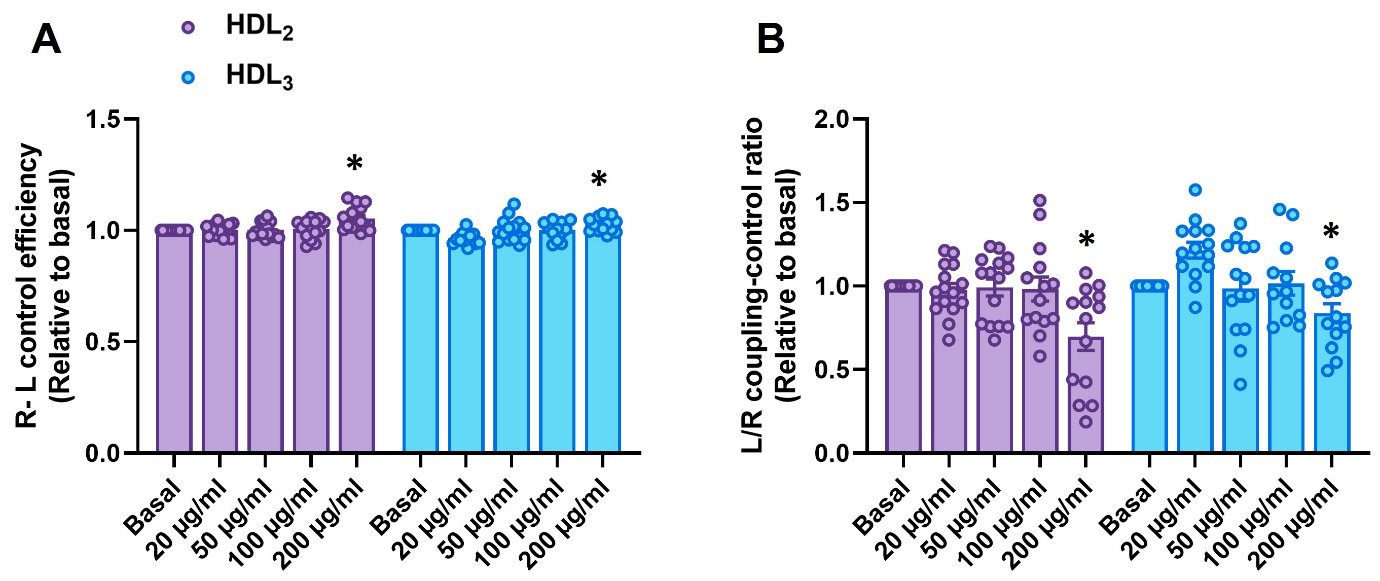
**

**S1 figure. Effects of human HDL_2_ and HDL_3_ on R-L control efficiency and L/R coupling control ratio of intact mouse myotubes in high glucose conditioning. (A)** R-L control efficiency and **(B)** L/R coupling-control ratio after 4 h treatment with or without human HDL_2_ or HDL_3_ present (12.5, 25, 50, 100, or 200 µg/ml) in high glucose (25 mmol/l) conditioning. Results are presented as means ± SEM relative to basal from 12-15 individual experiments (*n* = 12 for HDL_3_ 100 µg/ml; *n* = 13 for HDL_3_ 200 µg/ml; *n* = 14 for HDL_2_ 100 and 200 µg/ml, and HDL_3_ 20 and 50 µg/ml; *n* = 15 for basal, HDL_2_ 20 and 50 µg/ml in R-L control efficiency and L/R coupling-control ratio). *Statistically significant versus basal (*p* < 0.05, two-way ANOVA with Tukey’s post hoc comparison). Mean ± SEM of basal values: R-L control efficiency 0.85 ± 0.01; L/R coupling-control ratio 0.15 ± 0.01. R, basal respiration. L, proton leak.

**
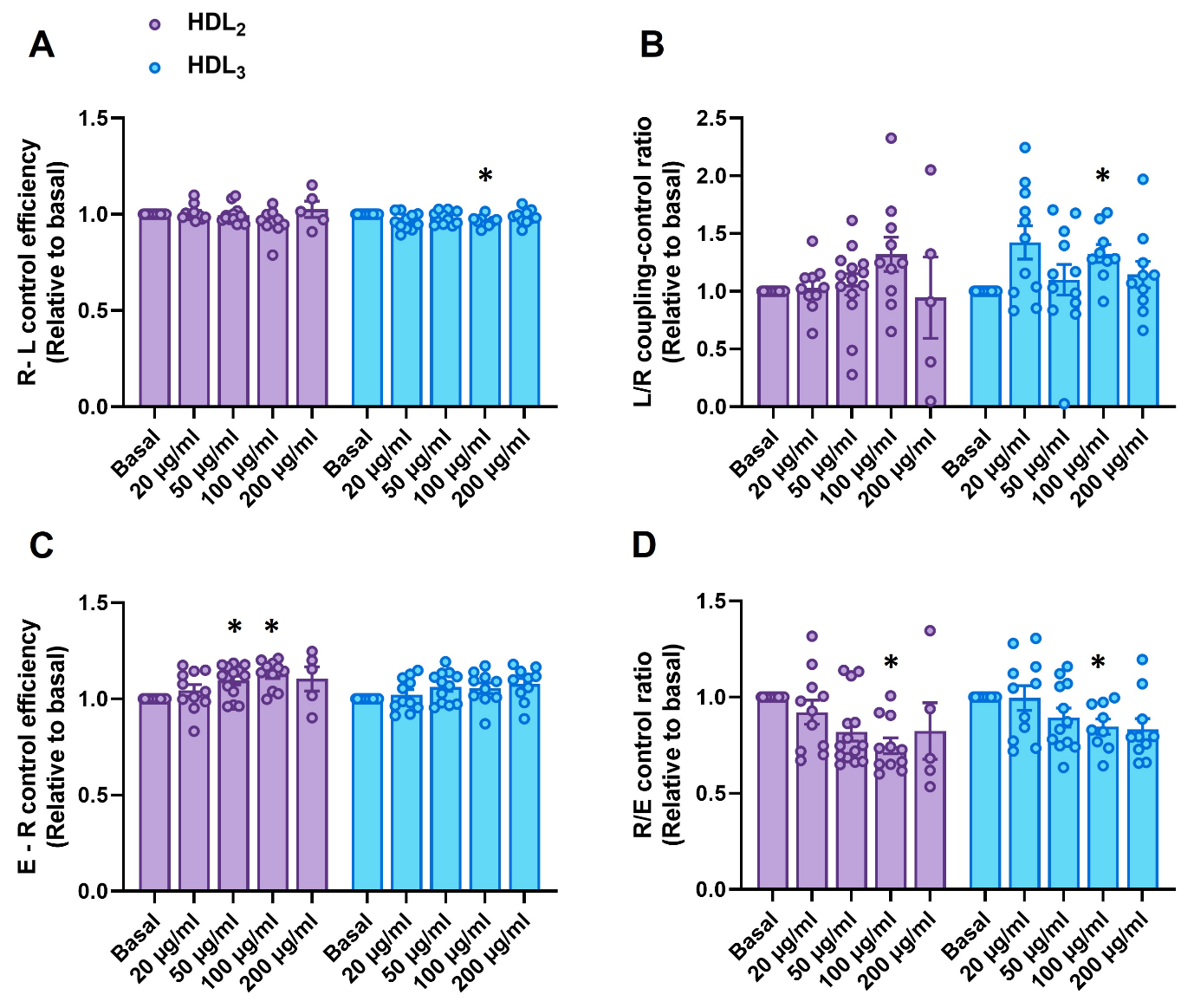
**

**S2 figure. Effects of human HDL_2_ and HDL_3_ on coupling control efficiencies of intact mouse myotubes in oleic acid conditioning. (A)** R-L control efficiency, **(B)** L/R coupling-control ratio, **(B)** E-R control efficiency, and **(D)** R/E control ratio after 4 h treatment with or without human HDL_2_ or HDL_3_ present (12.5, 25, 50, 100, or 200 µg/ml) in oleic acid (100 µmol/l albumin-bound oleic acid with 5.5 mmol/l glucose) conditioning. Results are presented as means ± SEM relative to basal from 5-14 individual experiments (*n* = 5 for HDL_2_ 200 µg/ml in R-L and E-R control efficiencies, L/R coupling-control ratio and R/E control ratio; *n* = 9 for HDL_3_ 100 µg/ml in R-L control efficiency and L/R coupling-control ratio; *n* = 10 for HDL_2_ 20 µg/ml in HDL_2_ 100 µg/ml in R-L control efficiency and L/R coupling-control ratio, HDL_3_ 100 µg/ml in E-R control efficiency and R/E control ratio, and HDL_3_ 200 µg/ml in R-L and E-R control efficiencies and L/R coupling-control ratio; *n* = 11 for basal, HDL_2_ 20 µg/ml and HDL_3_ 20 µg/ml in R-L and E-R control efficiencies and R/E control ratio, HDL_3_ 20 µg/ml in L/R coupling-control ratio, HDL_3_ 50 µg/ml in R-L control efficiency, HDL_2_ 100 µg/ml in E-R control efficiency, L/R coupling-control ratio and R/E control ratio, HDL_3_ 200 µg/ml in R/E control ratio; *n* = 12 for HDL_3_ 50 µg/ml in E-R control efficiency, L/R coupling-control ratio and R/E control ratio; *n* = 14 for HDL_2_ 50 µg/ml in E-R control efficiency, L/R coupling-control ratio and R/E control ratio). *Statistically significant versus basal (*p* < 0.05, two-way ANOVA with Tukey’s post hoc comparison). Mean ± SEM of basal values: R-L control efficiency 0.88 ± 0.01; L/R coupling-control ratio 0.12 ± 0.01; E-R control efficiency 0.69 ± 0.02; R/E control ratio 0.31 ± 0.02. R, basal respiration. L, proton leak. E, maximal respiration.

**
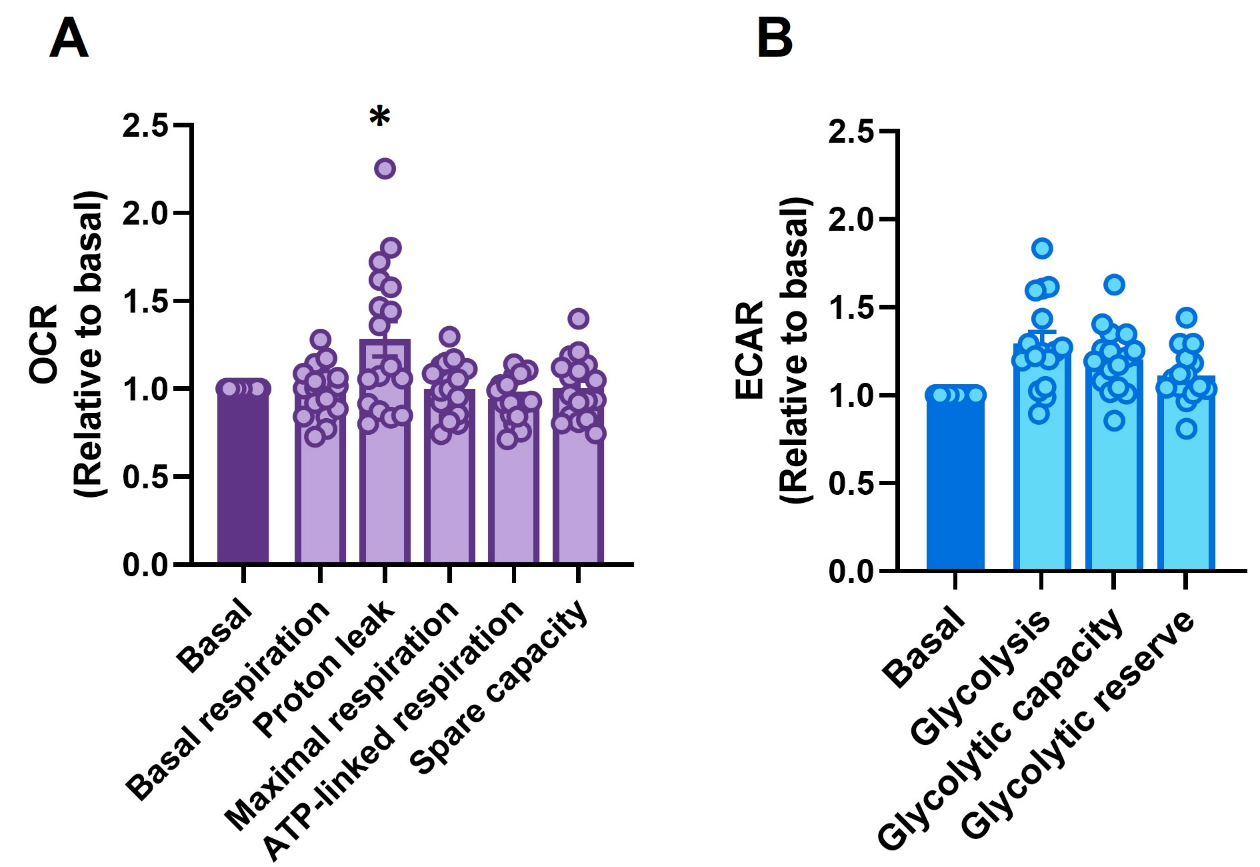
**

**S3 figure. Effect of human LDL on OCR and ECAR in mouse myotubes in high glucose conditioning.** (**A**) Cellular respiration parameters and (**B**) glycolytic flux parameters of mouse myotubes after 4 h treatment with or without 100 µg/ml of human LDL present in high glucose (25 mmol/l) conditioning. Results are presented as means ± SEM relative to basal from 16-17 individual experiments (*n* = 16 for each respiration state in OCR; *n* = 17 for each glycolytic flux parameter in ECAR). *Statistically significant versus basal (*p* < 0.05, one-way ANOVA with Tukey’s post hoc comparison). Mean ± SEM of basal values: basal respiration 4.6 ± 0.2 pmol/min/µg; proton leak 0.86 ± 0.03 pmol/min/µg; maximal respiration 16.9 ± 0.5 pmol/min/µg; ATP-linked respiration 3.7 ± 0.1 pmol/min/µg; spare capacity 12.3 ± 0.4 pmol/min/µg; glycolysis 1.6 ± 0.04 mpH/min/µg; glycolytic capacity 2.9 ± 0.09 mpH/min/µg; glycolytic reserve 1.3 ± 0.05 mpH/min/µg. OCR, oxygen consumption rate; ECAR, extracellular acidification rate.

**
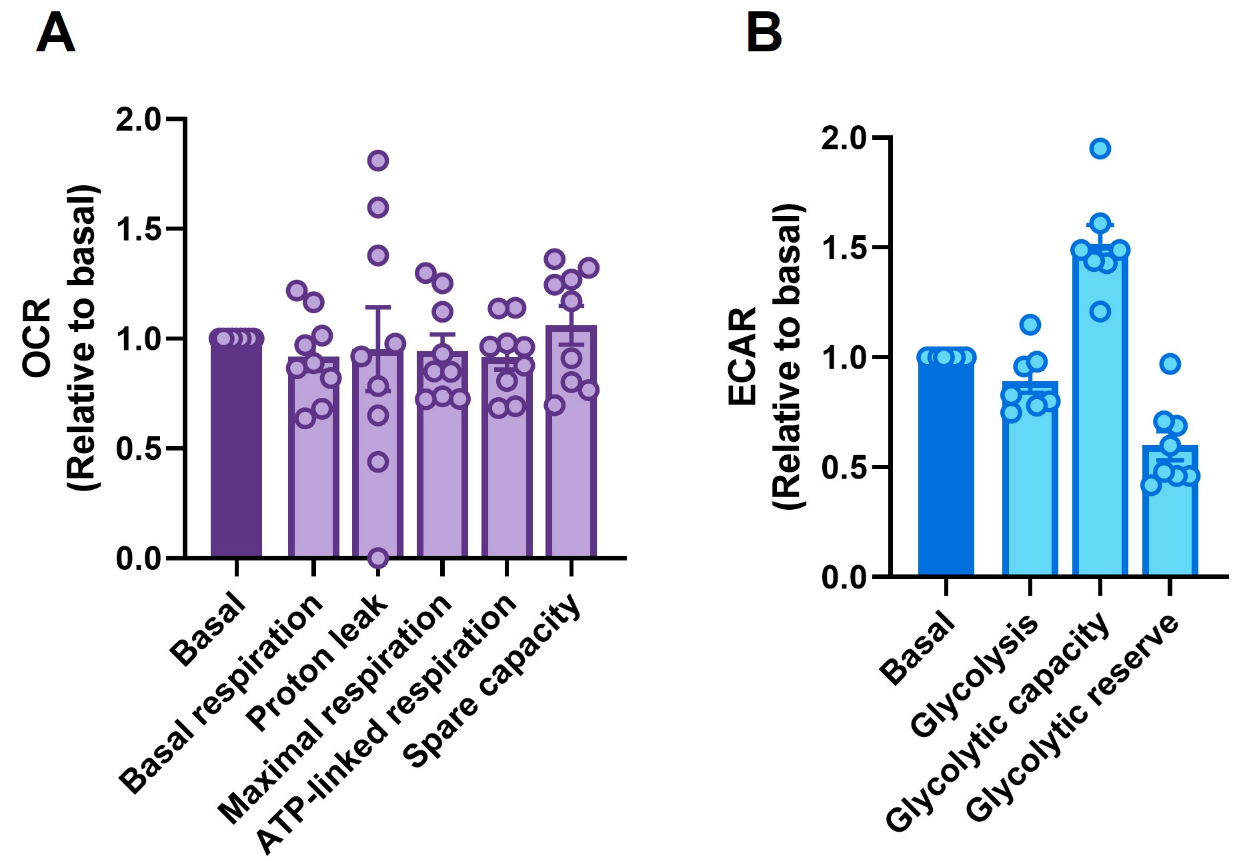
**

**S4 figure. No effect of human LDL on OCR and ECAR in mouse myotubes in oleic acid conditioning.** (**A**) Cellular respiration parameters and (**B**) glycolytic flux parameters of mouse myotubes after 4 h treatment with or without 100 µg/ml of human LDL present in oleic acid (100 µmol/l albumin-bound oleic acid with 5.5 mmol/l glucose) conditioning. Results are presented as means ± SEM relative to basal from 7-9 individual experiments (*n* = 7 for glycolysis and glycolytic capacity; *n* = 8 for glycolytic reserve; *n* = 9 for each respiration state in OCR). Statistical test, Kruskal-Wallis test with Dunn´s multiple comparisons test. Mean ± SEM of basal values: basal respiration 4.3 ± 0.2 pmol/min/µg; proton leak 0.36 ± 0.06 pmol/min/µg; maximal respiration 12.6 ± 0.6 pmol/min/µg; ATP-linked respiration 2.9 ± 0.1 pmol/min/µg; spare capacity 9.3 ± 0.6 pmol/min/µg; glycolysis 0.9 ± 0.05 mpH/min/µg; glycolytic capacity 1.5 ± 0.1 mpH/min/µg; glycolytic reserve 0.6 ± 0.07 mpH/min/µg. OCR, oxygen consumption rate; ECAR, extracellular acidification rate.

**
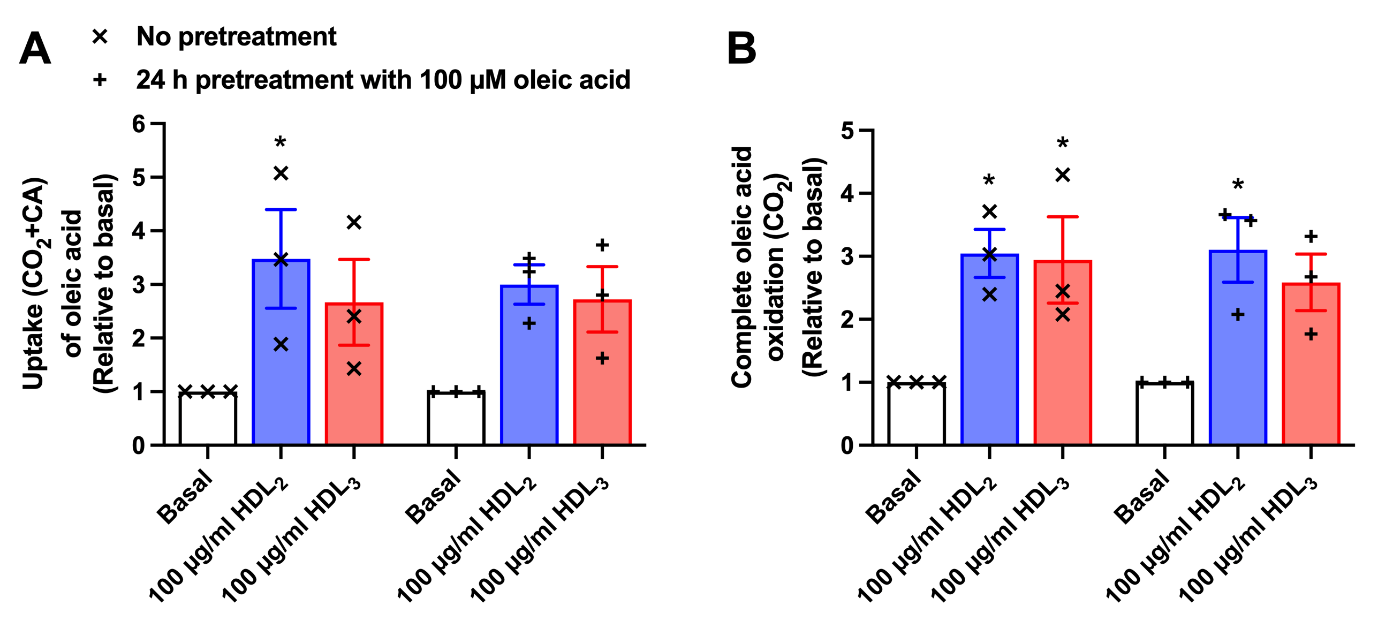
**

**S5 figure. Effect of human HDL_2_ and HDL_3_ on intracellular oleic acid metabolism.** The last 24 h of the differentiation period some culture wells were pretreated with 100 µmol/l unlabeled oleic acid. Thereafter, metabolism of 100 µmol/l [^14^C]oleic acid was examined in cultured human myotubes during 4 h incubation with or without 100 µg/ml of human HDL_2_ or HDL_3_ present. **(A)** Uptake (CO_2_+CA) and **(B)** complete oxidation (CO_2_) of oleic acid are shown. Results are presented as means ± SEM in nmol/mg protein from three individual experiments (*n* = 3). *Statistically significant versus basal (*p* < 0.05, one-way ANOVA with Dunnett´s correction). CA, cell-associated radioactivity.

**
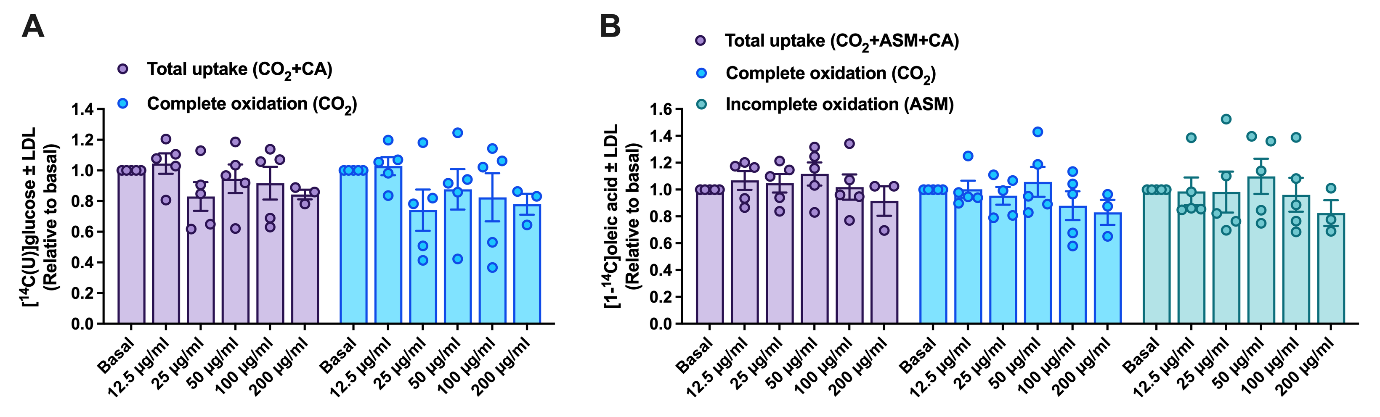
**

**S6 figure. No effect of human LDL on glucose and oleic acid metabolism.** Metabolism of **(A)** 200 µmol/l [^14^C]glucose or **(B)** 100 µmol/l [^14^C]oleic acid in cultured human myotubes after 4 h incubation with or without human LDL present (12.5, 25, 50, 100, or 200 µg/ml). Values are presented as means ± SEM from 3‑5 individual experiments (*n* = 5 for all experiments except for 200 µg/ml apoA-I which is based on *n* = 3). Statistical test, one-way ANOVA with Dunnett´s correction. Mean ± SEM of basal values: Total uptake: 36.2 ± 4.0 nmol/mg protein; complete oxidation: 15.4 ± 2.3 nmol/mg protein; incomplete oxidation: 95.9 ± 22.5 nmol/mg protein. CA, cell-associated radioactivity; ASM, acid-soluble metabolites.

**
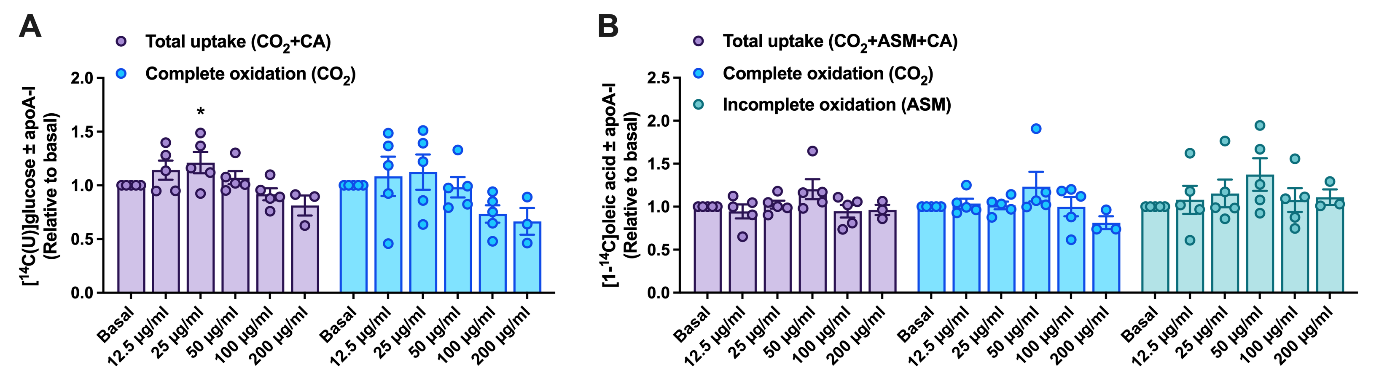
**

**S7 figure. Effect of apoA-I on glucose and oleic acid metabolism.** Metabolism of **(A)** 200 µmol/l [^14^C]glucose or **(B)** 100 µmol [^14^C]oleic acid in cultured human myotubes after 4 h incubation with or without apoA-I present (12.5, 25, 50, 100, or 200 µg/ml). Values are presented as means ± SEM from 3-5 individual experiments (*n* = 5 for all experiments except for 200 µg/ml apoA-I which is based on *n* = 3). *Statistically significant versus basal (*p* < 0.05, one-way ANOVA with Dunnett´s correction). CA, cell-associated radioactivity; ASM, acid-soluble metabolites.

**S1 table. Primer sequences.**

| Gene | Forward | Reverse |
| --- | --- | --- |
| *ABCA1* | GAA CAG CAG TTG GAT GGC TTA GA | ACA GAA CCA TTA CTG GAC TGG ACA T |
| *ABCG1* | TCG GGA ACG AAA CCA AGA AG | GAG GAA CAG CAT GGA GAA GAA GAG |
| *ACADM* | AGG CCG TGA CCC GTG TATT | AAC CCC GCT GCC ATG TT |
| *ANGPTL4* | CCT CCG CAG GGA CAA GAA | GTG GGA TGG AGC GGA AGT ACT |
| *ATP5F1B* | TGT GCC TGC TGA TGA CTT GAC | GTA GCA TCC AAA TGG GCA AAC |
| *ATP5MC2* | TGC GCA TGC TCG GAA AG | CGT GGA CTG CGG TTT GGT |
| *CD36* | AGT CAC TGC GAC ATG ATT AAT GGT | CTG CAA TAC CTG GCT TTT CTC AA |
| *COX5B* | CAA TGT ACT GGC CCC AAA GG | CAG CCT ACT ATT CTC TTG TTG GAG ATG |
| *CPT1B* | GAG GCC TCA ATG ACC AGA ATG | GTG GAC TCG CTG GTA CAG GAA |
| *GAPDH* | TGC ACC ACC ACC TGC TTA GC | GGC ATG GAC TGT GGT CAT GAG |
| *HK2* | CGG CTG CGC TCT ACT ATT GG | GTC TTA TGT AGA CGC TTG GCA AAA |
| *NDUFA8* | CGG AGA ATC CCT ATC ACT CAA GAC | GTG TGG CAG GCT GCA GAT CT |
| *PDK4* | TTT CCA GAA CCA ACC AAT TCA CA | TGC CCG CAT TGC ATT CTT A |
| *PPARGC1A* | AAA GGA TGC GCT CTC GTT CA | TCT ACT GCC TGG AGA CCT TGA TC |
| *RPLP0* | CCA TTC TAT CAT CAA CGG GTA CAA | AGC AAG TGG GAA GGT GTA ATC C |
| *SCARB1* | GTG GCT CCC AAA ACC CTG TT | ACG GGC AGA AGC CTT CGT |
| *SDHB* | TTG ACT CCA GAG ATG ACT TCA CAG A | GGT GTG GCA GCG GTA TAG AGA |
| *UQCRB* | AAG GTC CAA GGT CTC CTC TCT TC | CAT CTC CAG CAG GTA CTT CAC TCA |

**S2 table. Cellular respiration parameters without significant changes after human HDL_2_ and HDL_3_ treatment with high glucose conditioning in intact mouse myotubes.**

|  | Basal respiration  (*n* = 12 - 15) | Proton leak  (*n* = 12 - 15) | Maximal respiration  (*n* = 12 - 15) | Spare capacity  (*n* = 12 - 15) |
| --- | --- | --- | --- | --- |
| HDL_2_ (µg/ml) |  |  |  |  |
| 20 | 1.01 ± 0.05 | 0.99 ± 0.07 | 1.00 ± 0.04 | 0.99 ± 0.04 |
| 50 | 1.09 ± 0.07 | 1.07 ± 0.08 | 1.13 ± 0.05 | 1.15 ± 0.05 |
| 100 | 1.10 ± 0.05 | 1.09 ± 0.11 | 1.14 ± 0.06 | 1.15 ± 0.07 |
| 200 | 1.06 ± 0.08 | 0.79 ± 0.12 | 1.04 ± 0.05 | 1.03 ± 0.06 |
| HDL_3_ (µg/ml) |  |  |  |  |
| 20 | 1.08 ± 0.04 | 1.31 ± 0.09 | 1.10 ± 0.05 | 1.11 ± 0.05 |
| 50 | 1.10 ± 0.05 | 1.06 ± 0.09 | 1.11 ± 0.05 | 1.14 ± 0.07 |
| 100 | 1.17 ± 0.06 | 1.18 ± 0.11 | 1.30 ± 0.09 | 1.35 ± 0.11 |
| 200 | 1.15 ± 0.04 | 0.95 ± 0.07 | 1.13 ± 0.06 | 1.13 ± 0.07 |
| Basal (pmol/min/µg) | 4.61 ± 0.17 | 0.69 ± 0.06 | 17.00 ± 0.88 | 12.38 ± 0.76 |

Cellular respiration parameters from OCR data given as relative to basal (mean ± SEM) without significant changes (*p* > 0.05). The treatment was 4 h with human HDL_2_ and HDL_3_ (20, 50, 100, or 200 µg/ml) in high glucose (25 mmol/l) conditioning. Statistical test, two-way ANOVA with Tukey’s post hoc comparison. OCR, oxygen consumption rate.

**S3 table. Coupling-control efficiencies and FCRs without significant changes after human HDL_2_ and HDL_3_ treatment with high glucose conditioning in intact mouse myotubes.**

|  | E-L coupling control efficiency  (*n* = 12 - 15) | E-R control efficiency  (*n* = 12 - 15) | L/E coupling-control ratio  (*n* = 12 - 15) | R/E control ratio  (*n* = 12 - 15) |
| --- | --- | --- | --- | --- |
| HDL_2_ (µg/ml) |  |  |  |  |
| 20 | 1.000 ± 0.003 | 0.998 ± 0.011 | 1.001 ± 0.061 | 1.014 ± 0.031 |
| 50 | 1.002 ± 0.002 | 1.020 ± 0.010 | 0.948 ± 0.054 | 0.953 ± 0.026 |
| 100 | 1.001 ± 0.003 | 1.012 ± 0.010 | 0.956 ± 0.070 | 0.972 ± 0.026 |
| 200 | 1.011 ± 0.004 | 0.996 ± 0.018 | 0.729 ± 0.098 | 1.024 ± 0.048 |
| HDL_3_ (µg/ml) |  |  |  |  |
| 20 | 0.992 ± 0.002 | 1.010 ± 0.010 | 1.190 ± 0.055 | 0.976 ± 0.025 |
| 50 | 1.000 ± 0.004 | 1.017 ± 0.015 | 0.976 ± 0.096 | 0.962 ± 0.040 |
| 100 | 1.003 ± 0.003 | 1.034 ± 0.013 | 0.940 ± 0.078 | 0.919 ± 0.031 |
| 200 | 1.004 ± 0.003 | 0.995 ± 0.016 | 0.874 ± 0.080 | 1.025 ± 0.042 |
| Basal | 0.960 ± 0.002 | 0.723 ± 0.010 | 0.040 ± 0.002 | 0.277 ± 0.011 |

Coupling-control efficiencies and FCRs calculated from OCR data given as relative to basal (mean ± SEM) without significant changes (*p* > 0.05). The treatment was 4 h with human HDL_2_ and HDL_3_ (20, 50, 100, or 200 µg/ml) in high glucose (25 mmol/l) conditioning. Statistical test, two-way ANOVA with Tukey’s post hoc comparison. FCRs, flux control ratios; OCR, oxygen consumption rate; R, basal respiration; L, proton leak; E, maximal respiration.

**S4 table. Cellular respiration parameters without significant changes after human HDL_2_ and HDL_3_ treatment with oleic acid conditioning in intact mouse myotubes.**

|  | Proton leak  (*n* = 5 - 14) | ATP-linked respiration  (*n* = 5 - 13) | Maximal respiration  (*n* = 5 - 14) | Spare capacity  (*n* = 5 - 14) |
| --- | --- | --- | --- | --- |
| HDL_2_ (µg/ml) |  |  |  |  |
| 20 | 0.86 ± 0.15 | 0.83 ± 0.04 | 1.00 ± 1.10 | 1.04 ± 0.12 |
| 50 | 1.03 ± 0.12 | 0.92 ± 0.03 | 1.19 ± 0.07 | 1.31 ± 0.10 |
| 100 | 1.31 ± 0.15 | 0.98 ± 0.07 | 1.34 ± 0.10 | 1.51 ± 0.12 |
| 200 | 0.81 ± 0.30 | 0.84 ± 0.04 | 1.08 ± 0.16 | 1.22 ± 0.23 |
| HDL_3_ (µg/ml) |  |  |  |  |
| 20 | 1.39 ± 0.16 | 0.95 ± 0.05 | 1.03 ± 0.10 | 1.07 ± 0.13 |
| 50 | 1.13 ± 0.14 | 1.04 ± 0.05 | 1.20 ± 0.08 | 1.28 ± 0.11 |
| 100 | 1.07 ± 0.11 | 0.92 ± 0.05 | 1.06 ± 0.08 | 1.13 ± 0.10 |
| 200 | 1.14 ± 0.12 | 1.00 ± 0.07 | 1.24 ± 0.11 | 1.35 ± 0.15 |
| Basal (pmol/min/µg) | 0.46 ± 0.06 | 3.47 ± 0.17 | 13.49 ± 1.00 | 9.45 ± 0.96 |

Cellular respiration parameters from OCR data given as relative to basal (mean ± SEM) without significant changes (*p* > 0.05). The treatment was 4 h with human HDL_2_ and HDL_3_ (20, 50, 100, or 200 µg/ml) in oleic acid (100 µmol/l albumin-bound oleic acid with 5.5 mmol/l glucose) conditioning. Statistical test, two-way ANOVA with Tukey’s post hoc comparison. OCR, oxygen consumption rate.

**S5 table. Coupling-control efficiencies and FCRs without significant changes after human HDL_2_ and HDL_3_ treatment with oleic acid conditioning in intact mouse myotubes.**

|  | E-L coupling control efficiency  (*n* = 5 - 14) | L/E coupling-control ratio  (*n* = 5 - 14) |
| --- | --- | --- |
| HDL_2_ (µg/ml) |  |  |
| 20 | 1.006 ± 0.004 | 0.831 ± 0.135 |
| 50 | 1.007 ± 0.004 | 0.884 ± 0.109 |
| 100 | 1.001 ± 0.005 | 0.860 ± 0.175 |
| 200 | 1.013 ± 0.014 | 0.957 ± 0.483 |
| HDL_3_ (µg/ml) |  |  |
| 20 | 0.994 ± 0.005 | 1.481 ± 0.232 |
| 50 | 1.004 ± 0.005 | 1.014 ± 0.167 |
| 100 | 1.003 ± 0.009 | 1.067 ± 0.225 |
| 200 | 1.004 ± 0.004 | 0.854 ± 0.092 |
| Basal | 0.963 ± 0.005 | 0.037 ± 0.005 |

Coupling-control efficiencies and FCRs calculated from OCR data given as relative to basal (mean ± SEM) without significant changes (*p* > 0.05). The treatment was 4 h with human HDL_2_ and HDL_3_ (20, 50, 100, or 200 µg/ml) in oleic acid (100 µmol/l albumin-bound oleic acid with 5.5 mmol/l glucose) conditioning. Statistical test, two-way ANOVA with Tukey’s post hoc comparison. OCR, oxygen consumption rate; L, proton leak; E, maximal respiration.

**S6 table. Glucose oxidative metabolism and fatty acid oxidation without significant changes after human HDL_2_ and HDL_3_ treatment in permeabilized mouse myotubes.**

|  | Glucose oxidative metabolism | | | Fatty acid oxidation | |
| --- | --- | --- | --- | --- | --- |
|  | LEAK  (*n* = 7 - 8) | OXPHOS I + II  (*n* = 8) | ETS  (*n* = 7 - 8) | LEAK  (*n* = 7 - 8) | ETS  (*n* = 8) |
| HDL_2_  (100 µg/ml) | 1.65 ± 0.39 | 1.25 ± 0.16 | 1.32 ± 0.18 | - 0.41 ± 1.42 | 1.85 ± 0.37 |
| HDL_3_  (100 µg/ml) | 1.52 ± 0.21 | 1.25 ± 0.11 | 1.19 ± 0.09 | 0.43 ± 0.50 | 1.25 ± 0.14 |
| Basal  O_2_ pmol/((s x ml)/(U/ml)) | 257 ± 41 | 4286 ± 354 | 5716 ± 584 | 53 ± 79 | 1338 ± 53 |

Mitochondrial respiration states given as relative to basal (mean ± SEM) without significant changes (*p* > 0.05). Substrates injected to induce different mitochondrial respiration states after permeabilization in glucose oxidative metabolism: pyruvate (5 mmol/l) and malate (2 mmol/l) for OXPHOS I and LEAK, and ADP + Mg^2+^ (5 mmol/l + 3 mmol/l), glutamate (10 mmol/l) and succinate (10 mmol/l) for OXPHOS I+II and ETS. Substrates injected to induce different mitochondrial respiration states after permeabilization in fatty acid oxidation: malate (0.1 mmol/l) for LEAK, ADP + Mg^2+^ (5 mmol/l + 3 mmol/l) and palmitoylcarnitine (10 µmol/l) for CI+FAO and ETS. Statistical test, one-way ANOVA with Tukey’s post hoc comparison and Kruskal-Wallis test with Dunn´s multiple comparisons test.

**S7 table. Coupling-control efficiencies and FCRs of glucose oxidative metabolism without significant changes after human HDL_2_ and HDL_3_ treatment in permeabilized mouse myotubes.**

|  | HDL_2_ (100 µg/ml) | HDL_3_ (100 µg/ml) | Basal |
| --- | --- | --- | --- |
| P-L control efficiency (*n* = 7 – 8) | 0.980 ± 0.011 | 0.982 ± 0.020 | 0.939 ± 0.012 |
| E-L coupling efficiency (*n* = 8) | 0.986 ± 0.008 | 0.992 ± 0.020 | 0.951 ± 0.011 |
| E-P control efficiency (*n* = 7) | 1.074 ± 0.108 | 1.181 ± 0.188 | 0.220 ± 0.023 |
|  |  |  |  |
| L/P coupling-control ratio (*n* = 7 – 8) | 1.300 ± 0.258 | 1.244 ± 0.200 | 0.089 ± 0.015 |
| L/E coupling-control ratio (*n* = 7 – 8) | 1.283 ± 0.255 | 1.144 ± 0.197 | 0.049 ± 0.011 |
| P/E control ratio (*n* = 8) | 0.984 ± 0.054 | 0.948 ± 0.058 | 0.534 ± 0.032 |

Coupling-control efficiencies and FCRs calculated from mitochondrial respiration states given as relative to basal (mean ± SEM) without significant changes (*p* > 0.05). Substrates injected to induce different mitochondrial respiration states after permeabilization in glucose oxidative metabolism: pyruvate (5 mmol/l) and malate (2 mmol/l) for OXPHOS I and LEAK, and ADP + Mg^2+^ (5 mmol/l + 3 mmol/l), glutamate (10 mmol/l) and succinate (10 mmol/l) for OXPHOS I+II and ETS. Statistical test, one-way ANOVA with Tukey’s post hoc comparison and Kruskal-Wallis test with Dunn´s multiple comparisons test. P, OXPHOS I; L, LEAK; E, ETS.

**S8 table. Coupling-control efficiencies and FCRs of fatty acid oxidation without significant changes after human HDL_2_ and HDL_3_ treatment in permeabilized mouse myotubes.**

|  | HDL_2_ (100 µg/ml) | HDL_3_ (100 µg/ml) | Basal |
| --- | --- | --- | --- |
| P-L control efficiency (*n* = 8) | 0.903 ± 0.086 | 1.002 ± 0.092 | 0.964 ± 0.086 |
| E-L coupling efficiency (*n* = 8) | 0.922 ± 0.060 | 0.984 ± 0.062 | 0.968 ± 0.058 |
| E-P control efficiency (*n* = 8) | 0.930 ± 0.272 | 0.960 ± 0.125 | 0.253 ± 0.039 |
|  |  |  |  |
| L/P coupling-control ratio (*n* = 7 – 8) | 0.250 ± 0.611 | -0.137 ± 0.537 | 0.036 ± 0.086 |
| L/E coupling-control ratio (*n* = 8) | -0.443 ± 0.907 | -0.140 ± 0.561 | 0.032 ± 0.058 |
| P/E control ratio (*n* = 8) | 1.083 ± 0.073 | 1.038 ± 0.052 | 0.747 ± 0.039 |

Coupling-control efficiencies and FCRs calculated from mitochondrial respiration states given as relative to basal (mean ± SEM) without significant changes (*p* > 0.05). Substrates injected to induce different mitochondrial respiration states after permeabilization in fatty acid oxidation: malate (0.1 mmol/l) for LEAK, ADP + Mg^2+^ (5 mmol/l + 3 mmol/l) and palmitoylcarnitine (10 µmol/l) for CI+FAO and ETS. Statistical test, one-way ANOVA with Tukey’s post hoc comparison. P, OXPHOS I; L, LEAK; E, ETS.
